# Exploring the Needs and Requirements of Informal Caregivers of Older Adults With Cognitive Impairment From Sensor-Based Care Solutions: Multimethod Study

**DOI:** 10.2196/49319

**Published:** 2023-10-25

**Authors:** Nikita Sharma, Louise M A Braakman-Jansen, Harri Oinas-Kukkonen, Jan Hendrik Croockewit, JEWC van Gemert-Pijnen

**Affiliations:** 1 Faculty of Behavioural, Management and Social Sciences University of Twente, Enschede Enschede Netherlands; 2 Faculty of Information Technology and Electrical Engineering University of Oulu Oulu Finland; 3 NEDAP N.V. Groenlo Netherlands

**Keywords:** informal caregiving, cognitive impairment, unobtrusive sensing solutions, in-home care, aging in place, assistive technologies

## Abstract

**Background:**

With the increase in the older adult population, sensor-based care solutions that can monitor the deviations in physical, emotional, and physiological activities in real-time from a distance are demanded for prolonging the stay of community-dwelling older adults with cognitive impairment. To effectively develop and implement these care solutions, it is important to understand the current experiences, future expectations, perceived usefulness (PU), and communication needs of the informal caregivers of older adults with cognitive impairment regarding such solutions.

**Objective:**

This comprehensive study with informal caregivers of older adults with cognitive impairment aims to (1) highlight current experiences with (if any) and future expectations from general sensor-based care solutions, (2) explore PU specifically toward unobtrusive sensing solutions (USSs), (3) determine the information communication (IC) needs and requirements for communicating the information obtained through USSs in different care scenarios (fall, nocturnal unrest, agitation, and normal daily life), and (4) elicit the design features for designing the interaction platform in accordance with the persuasive system design (PSD) model.

**Methods:**

A multimethod research approach encompassing a survey (N=464) and in-depth interviews (10/464, 2.2%) with informal caregivers of older adults with cognitive impairment was used. The insights into past experiences with and future expectations from the sensor-based care solutions were obtained through inductive thematic analysis of the interviews. A convergent mixed methods approach was used to explore PU and gather the IC needs from USSs by using scenario-specific questions in both survey and interviews. Finally, the design features were elicited by using the PSD model on the obtained IC needs and requirements.

**Results:**

Informal caregivers expect care infrastructure to consider centralized and empathetic care approaches. Specifically, sensor-based care solutions should be adaptable to care needs, demonstrate trust and reliability, and ensure privacy and safety. Most informal caregivers found USSs to be useful for emergencies (mean 4.09, SD 0.04) rather than for monitoring normal daily life activities (mean 3.50, SD 0.04). Moreover, they display variations in information needs including mode, content, time, and stakeholders involved based on the care scenario at hand. Finally, PSD features, namely, reduction, tailoring, personalization, reminders, suggestions, trustworthiness, and social learning, were identified for various care scenarios.

**Conclusions:**

From the obtained results, it can be concluded that the care scenario at hand drives PU and IC design needs and requirements toward USSs. Therefore, future technology developers are recommended to develop technology that can be easily adapted to diverse care scenarios, whereas designers of such sensor-driven platforms are encouraged to go beyond tailoring and strive for *strong personalization* while maintaining the privacy of the users.

## Introduction

### Background

The global population of older adults with cognitive impairment who live alone is increasing tremendously. This demographic shift necessitates the implementation of advanced sensor-based care solutions to support both individuals with cognitive impairment and their caregivers [1,2]. The advanced solutions having the ability to monitor 24/7 can not only provide peace of mind to caregivers but are also advantageous in optimizing care by using long-term monitored information. Thus, they appear more promising in prolonging the stay of community-dwelling older adults with cognitive impairment who are living alone and receiving home care [3].

Broadly, any sensor-based monitoring solution has 3 parts: a physical sensing unit, a computing unit, and an interaction unit. The physical sensing unit deployed in the homes of older adults with cognitive impairment is responsible for collecting data related to the person being monitored or the surrounding environment. These collected sensing data are then processed by the computing unit, which typically uses machine learning algorithms to extract meaningful insights regarding the activities, behaviors, and well-being of the person being monitored. Finally, the communicating unit, often in the form of an eHealth app, serves as a channel of communication between the sensing system and the caregivers or other stakeholders involved in the care of older adults with cognitive impairment. It facilitates real-time communication and collaboration among caregivers, ensuring timely information exchange and coordinated care efforts for the benefit of the care recipient (CR). For example, the My Guardian watch can support older adult care by providing alerts or notifications for day-to-day tasks to the older adult, whereas caregivers can monitor them from a distance (such as tracking their location, etc) via an app [4]. The watch can be seen as a sensing unit coupled with a computing unit collecting data from the older adult and performing the necessary computations to generate insights. The app, developed on top of this system, acts as an interaction platform, responsible for communicating the information obtained by the watch from the older adults to the informal caregivers to enable care from a distance.

A variety of in-home, sensor-based monitoring solutions for older adult care are available [5] such as wearable systems (eg, fall alarm pendants and medical guardians) [6], vision-based systems (eg, nest cam and ring indoor cam) [7], and device-free sensing systems (eg, Sensara) [1]. Literature has delineated the disadvantages of camera-based and wearable solutions in the care of older adults with cognitive impairment; the former enables monitoring only in the line of sight and is prone to privacy issues, whereas the latter mandates the continuous wearing of the device for continued monitoring. Nevertheless, device-free sensing systems overcome the abovementioned disadvantages, as they are unobtrusive in nature and do not impose an active region of operation, making them more suitable for the care of older adults with cognitive impairment [1].

In this study, first, we aimed to cross-examine the experiences (both positive and negative), if any, of informal caregivers of the older adults with cognitive impairment who use sensor-based monitoring solutions (such as cameras, wearables, or device-free systems) in their caregiving practices. The insights into current experiences can facilitate the informed development of novel sensing solutions. Along with experiences, the expectations of informal caregivers from future care solutions were highlighted [8]. The expectations can inspire technology developers and designers by anticipating the attitudes, demands, and challenges that informal caregivers face on a daily basis [3]. For example, informal caregivers expect to attain peace of mind by ensuring the safety of the CR via care solution, but their experiences regarding privacy, reliability, and usability might vary with respect to the sensing solution at hand.

Currently, considerable efforts are made in the direction of exploring novel sensing technologies for unobtrusive or device-free sensing (such as Wi-Fi, mmWave, etc) [9-11]. These unobtrusive sensing solutions (USSs) can be defined as solutions that do not draw the user’s (in this case, older adults) attention or demand their direct involvement while blending well with the surroundings [1]. For example, Wi-Fi signals (which can be imagined as invisible waves traveling through the air that get affected by the surrounding movements) carry sensing information about the surrounding environment (even beyond the wall) [12], which can be analyzed using machine learning algorithms to detect human activities such as fall, continuous sitting and standing, gestures or behaviors such as emotional states, sleeping patterns, and so on. [13,14]. Therefore, they are found to be more compatible with older adult care [15]. In this study, we use the term “unobtrusive” specifically to define the proposed technology and do not extend it to information communication (IC) with other stakeholders.

In the past decade, rapid growth in developing USSs has been noticed [16,17], making it necessary to obtain a broad view of their implementation prospect. Therefore, the second objective of this study was to understand the perceived usefulness (PU) of USSs among informal caregivers when deployed in the care of older adults with cognitive impairment. Along with the development of USSs for robust performance (such as the use of deep neural networks to minimize false alarms), studies to understand the IC needs of informal caregivers toward USSs to provide them with optimal and on-time care information is required. USSs are intended to monitor older adults with cognitive impairment continuously, leading to a substantial amount of monitoring data that could be overwhelming or can cause information overload, if directly communicated to informal caregivers [18-20]. Therefore, it is crucial to optimize IC by considering the needs and requirements of informal caregivers of older adults with cognitive impairment in diverse care scenarios. For example, informal caregivers expect to be notified immediately during an emergency such as a fall, whereas they might not want to receive notification for nocturnal unrest immediately. Therefore, the third objective of this study was to determine the IC needs and requirements of informal caregivers for 4 care scenarios including emergencies (falls), short-term monitoring (nocturnal unrest), long-term monitoring (agitation), and normal daily life. Finally, to facilitate the design process of such an interaction platform, the design features were elicited based on the obtained IC needs and requirements in accordance with the persuasive system design (PSD) model [21].

### Research Objectives

In summary, this study had four research objectives:

1. To cross-examine the current experiences, if any, and highlight the future expectations from sensor-based care solutions; the knowledge about current experiences can inform the development of new sensing solutions, and knowledge about future expectations can help in anticipating the potential benefits and challenges that may arise during the deployment of USSs
2. To explore the PU of USSs among informal caregivers; exploring the PU during the development stage can inform the technology developers and designers about the possible acceptance of the developed technology
3. To determine the IC needs and requirements to design an interaction platform for communicating the information obtained through unobtrusive sensing systems in different care scenarios including fall, nocturnal rest, agitation, and normal daily life among informal caregivers of older adults with cognitive impairment and living alone
4. To elicit the *design features* based on the obtained needs and requirements for designing the interaction platform in accordance with the PSD model

## Methods

### Ethical Considerations

The Ethics Committee of the Behavioral, Management, and Social Sciences at the University of Twente granted formal ethical approval for the execution of this study (request 220250). Prior to participation in surveys and interviews, all participants were presented with a comprehensive oral or written description outlining the study's objectives, methodologies, data collection procedures, storage protocols, and the intended use of the collected data. Subsequently, a signed consent form was obtained from each participant. Participants were assured of their right to withdraw from the survey or interview at any stage if they felt uncomfortable.

In the survey, no personal details of the participants were collected to maintain their anonymity. However, it is essential to note that the surveyed individuals were users of the Caren platform. Consequently, obtaining additional permission from NEDAP N.V. will be imperative for any secondary analysis of the data collected. For interview data, which included intricate personal information about the care recipients and their corresponding informal caregivers, de-identification was done before analysis, ensuring confidentiality and privacy. If required, a summary of the qualitative data can be made available upon request. Lastly, as a token of appreciation for their valuable time and contributions, a small honorarium was provided to the participants.

### Study Design

Overall, a multimethod design encompassing survey (quantitative) and in-depth interview (qualitative) was used. Particularly, the survey was conducted on the Caren platform (NEDAP Healthcare) [22], a digital caregiving platform widely used by informal caregivers, CRs, and occasionally formal caregivers in the Netherlands to gain insights into their own health or the health of their CR. The Caren platform has no restrictions based on age group or type of illness.

**Figure 1:**
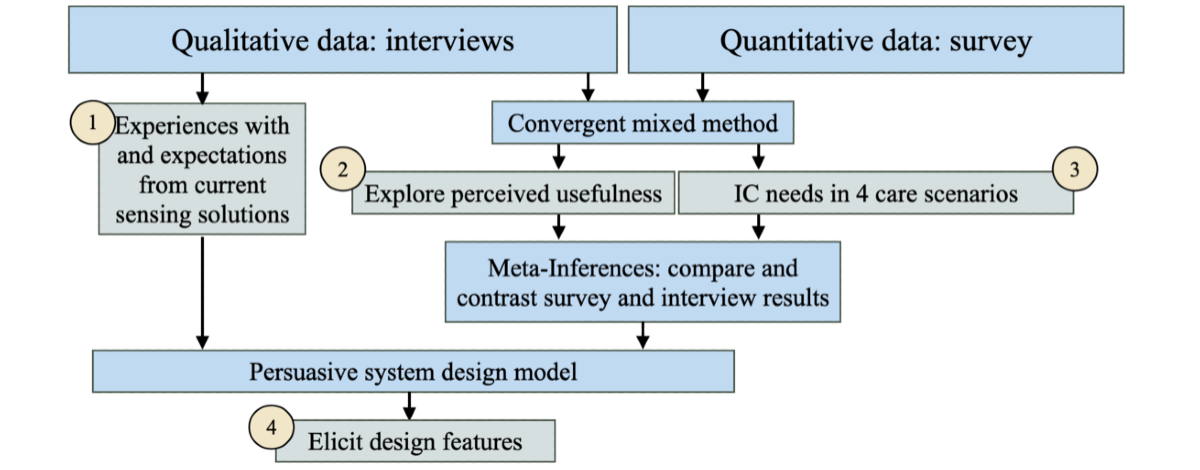
Study design depicting the 4 research objectives undertaken in this comprehensive study. IC: information communication.

The survey itself had two objectives: (1) to gather feedback about the use of the Caren platform from all users (CRs, informal caregivers, and formal caregivers) and (2) to specifically investigate the IC needs and requirements of informal caregivers. To achieve these objectives, the survey included questions about the use of the Caren platform (constructed by the Caren development team) and was presented to all users active during the survey duration (7 days). However, users who identified themselves as informal caregivers were shown questions related to PU and IC needs. Furthermore, informal caregivers who participated in the survey and expressed interest in further caregiving-related studies were invited for interviews. The methodologies used to address each research objective are as follows:

1. For *objective 1*, insights into past experiences and future expectations regarding sensor-based care solutions were obtained through interviews (Figure 1).
2. To address *objectives 2 and 3*, a convergent mixed methods approach was used. Scenario-specific questions, based on previous study [2], were used to gather IC needs from informal caregivers. This approach allowed for the combination of different methods, overcoming individual weaknesses and facilitating comparison, validation, and identification of any contradictions in the results. Meta-inferences were derived as a result [23] (Figure 1).
3. *Objective 4* aimed to elicit design features, which were derived using the PSD model based on the meta-inferences drawn from the IC needs and requirements of informal caregivers (Figure 1).

### Participants

A total of 6934 responses, including 1289 (18.59%) from self-managing CRs, 5583 (80.52%) from informal caregivers, and 62 (0.89%) from formal caregivers using the Caren platform were recorded. As the focus of the study was specifically on identifying the IC needs and requirements of informal caregivers, responses regarding the feedback about the Caren platform from all users were excluded from the analysis.

**Figure 2:**
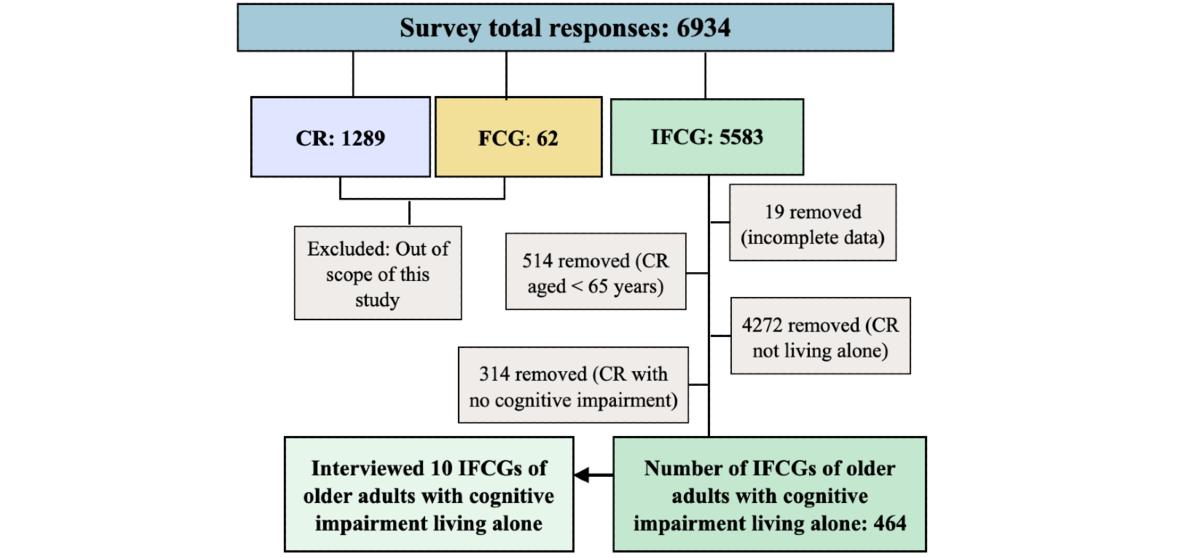
Participant sampling procedure for survey and interviews. CR: care recipient; FCG: formal caregiver; IFCG: informal caregiver.

The inclusion criteria for this study were as follows: (1) should be informal caregivers (friends or family) providing unpaid care, (2) should provide care for older adults (aged ≥65 y) who have cognitive impairment (can be mild or high), and (3) older adults with cognitive impairment should be living alone in their own homes. Purposive sampling was performed on the 80.52% (5583/6934) of responses of informal caregivers to select those who met these criteria. Initially, we excluded 0.34% (19/5583) of incomplete responses from informal caregivers who dropped out of the survey before completion or did not respond to mandatory questions. Thereafter, responses from informal caregivers who care for individuals aged <65 years (514/5583, 9.2% of responses) and do not live alone (4272/5583, 76.51% of responses) and whose CRs show no signs of cognitive impairment (314/5583, 5.62% of responses) were also excluded from the analysis. This resulted in a final sample size of 8.31% (464/5583) of responses. In addition, 10 informal caregivers were recruited for interviews from the same sample. Figure 2 illustrates the sample selection process from the survey data and for the interviews.

Finally, it is important to highlight that the indication of cognitive impairment in this study was solely based on the observations and indications provided by informal caregivers. Formal diagnoses of cognitive impairment are not always available unless the condition has reached an advanced stage. In such cases, informal caregivers serve as the primary identifiers of initial symptoms and changes indicative of cognitive decline [24,25]. Therefore, in this study, we also gave significant value to the personal experiences and insights of informal caregivers.

### Materials

#### Survey

The survey was used for gaining quantitative insights into the possible needs and requirements of informal caregivers of older adults with cognitive impairment toward a sensor-dependent interaction platform. In the survey, participants’ demographics (age and sex) and care elements information (age of informal caregiver and CR, sex, relationship with the CR, and the number of CRs they provide care to) were recorded. PU was measured with the help of 3 questions from the PU (Cronbach α=.898) construct of Technology Acceptance Model 2 [26] using a 7-point Likert scale (1=strongly disagree to 7=strongly agree). Given the exploratory stage of technology (Technology Readiness Level 2/4) [27], only a subset of 3 questions from the PU construct was used to obtain a broad view about its usefulness. However, additional customized questions were used to gain a more comprehensive understanding of changes in PU, if any, owing to different care scenarios (monitoring daily life activities and emergencies) and the stakeholders involved (themselves and their CRs) by using a 5-point Likert scale (1=strongly disagree to 5=strongly agree).

Furthermore, to identify the needs and requirements of informal caregivers, different care scenarios, namely, fall, nocturnal unrest, agitation, and normal daily life, were defined. These care scenarios were selected based on previous studies that highlighted important monitoring goals for USSs in 4 categories: safety (fall), health related (hygiene), psychological (nocturnal unrest), and psychosocial (agitation) [2]. Each of these scenarios contained custom-designed questions that were developed by the involved researchers’ team comprising various stakeholders in older adult care such as care platform designers or managers, design or eHealth researchers, and experts in the field. This approach was adopted owing to the absence of a standardized questionnaire in the existing literature addressing the IC needs of informal caregivers. The developed questionnaire underwent refinement and face validation through feedback from informal caregivers before its launch to ensure its appropriateness and relevance.

All scenarios had 5 similar questions: type of situation (what: emergency, acute, or normal), mode of IC (how: voice call, notification, SMS text message, email, or self-check), timing of IC (when: immediately, after a few minutes, or self-check), content of the information (what: raw data, interpreted, or interpreted with suggestion), and intended stakeholder or recipient of the information (whom: formal caregivers, wait for primary informal caregiver’s response, secondary informal caregivers, ambulance, self-check, or no information). Here, “raw data” refers to data directly obtained from sensors (such as numbers), for example, “Mr. X fall, and his heart rate is 120bpm.” “Interpreted data” means raw data that are further processed to make them more intuitive and insightful for informal caregivers to understand, for example, “Mr. X fall in the bathroom and his heart rate is higher than normal, which could indicate a heart attack.” The “interpretation with suggestion” option provides suggestions along with the interpretation, so that informal caregivers can make informed decisions, for example, “Mr. X fall in the bathroom and his heart rate is high. You might consider visiting him as soon as possible and informing the doctors.” Here, the preferred choice or choices for the respective questions were recorded.

In addition, for the scenarios of nocturnal unrest and agitation, a question was included to obtain the preference for the need for a detailed report (every day to informal caregivers, observe for a few days or weeks and then send to informal caregivers or formal caregivers, or no reports required). Participants were also asked for the possibility to provide feedback (yes, no, or maybe) about improving the system, considering the possibility of false alarms. Furthermore, a question was included to assess the need for providing compliments for the care tasks they perform (yes, no, or maybe).

It should be noted that all participants were asked to answer about normal daily life scenario, and they can choose 1 relevant care scenario among falls, nocturnal unrest, and agitation. Table 1 provides an overview of these questions. The English version of the survey questions can be found in Multimedia Appendix 1.

**Table 1:** Overview of the questions used in the survey for identifying the information communication (IC) needs.

Questions	Options
Type of situation	Emergency, acute, and normal
Time (when) of IC	Immediately, few minutes after, and self-check
Mode (how) of IC	Voice call, notification, SMS, email, and self-check
Care information recipient (whom) along with primary caregiver	Formal caregivers, wait for primary informal caregiver’s response, secondary informal caregivers, emergency services (ambulance), self-check, and no information
Content (what) of the information	Raw, interpreted, interpreted with suggestion, and others
Need of detailed report for nocturnal unrest and agitation scenarios	Every day to informal caregivers, observe for a few days and then send to informal caregivers, observe for a few weeks and then send to informal caregivers, send reports to formal caregiver, and no reports required
Feedback to improve USS^a^	Yes, maybe, and no
Compliment for the care tasks undertaken	Yes, maybe, and no

^a^USS: unobtrusive sensing solution.

#### Interviews

Semistructured interviews were conducted with 3 objectives: first, to highlight the current experiences and future expectations from sensor-based solutions; second, to gain an overview of PU (codefined with the survey); and third, to determine the IC needs and requirements of informal caregivers toward the interaction platform for different care scenarios (codefined with the survey).

Open-ended questions were asked about their experience with current technology use in facilitating care, with a focus on possible bottlenecks they face on a daily basis with these systems or in general care tasks. Then, questions were asked to understand their expectations from in-general care infrastructure and care solutions. To explore PU, their opinions about the use of USSs in different care scenarios by different stakeholders and in-general value of the system in organizing care (positive or negative) were discussed.

In-depth questions were asked about the 4 care scenarios including mode (how), time (when), content (what), information-receiving stakeholder (whom), feedback to the system, and need for compliments (consistent with survey questions and as shown in Table 1). In addition, they were asked about the influence of reporting the prediction of the system in the form of confidence percentage, such that, when the system is not very confident in the output but is suspicious of certain activity, it can indicate its confidence in the prediction as a percentage, for example, “System is 20% sure that your care recipient has fallen.” Finally, the participants’ opinions about adding social support to the interaction platform to facilitate communication between similar user groups were discussed. Multimedia Appendix 2 lists the questions asked in the interviews.

### Procedure

#### Survey

At the beginning of the survey, the idea of a USS or device-free sensing, specifically Wi-Fi Channel State Information (CSI), was introduced with the help of pictures depicting the placement of the sensor in the corner of the house, facilitating the detection of normal and fall situations (Multimedia Appendix 1). These pictures were inspired by previous studies of using CSI for detecting human activities [28]. Thereafter, web-based consent was obtained from the participants. Then, participants’ demographics and care elements information were recorded. Finally, questions about PU and design needs regarding the interaction platform were posed. All the survey questions were adapted in the Dutch language as it was conducted in the Netherlands. The overall completion time for the survey was approximately 15 to 20 minutes.

#### Interviews

At the beginning of the interviews, informal caregivers were provided with an oral and written explanation about the aims of the interview and USSs. Upon obtaining their consent, demographics and care elements (consistent with the survey) were asked. Thereafter, open-ended questions about current experience and future expectations were asked. Semistructured questions regarding PU specific to USSs (Wi-Fi CSI based) were posed. Then, a simple prototype showing a few screens of the interaction platform was presented to participants to provide them with a basic idea of the interaction platform (Multimedia Appendix 2). Participants were then asked to choose and answer for 1 scenario from a list of care scenarios including falls, nocturnal unrest, and agitation. Comparative questions were posed about normal daily life when no anomalies are detected, to understand the IC needs during a normal day in care. The interviews were conducted in Dutch by a native Dutch speaker and were audio recorded to facilitate analysis. The duration of the interviews was approximately 60 to 75 minutes. Overall, the information obtained from 10 interviews was found to be optimal, and no further interviews were conducted.

### Data Analysis

#### Survey

SPSS (version 28.0.1.0; IBM Corp) was used for analyzing the survey data. First, data corresponding to the included sample, that is, informal caregivers of older adults with cognitive impairment and living alone, were extracted. Descriptive statistics were used to analyze participants’ demographics (age and sex), care elements (age of CR, relationship with the CR, and number of CRs), response to questions corresponding to PU, and IC needs for different monitoring scenarios (fall, agitation, nocturnal unrest, and normal daily life).

#### Interviews

The interviews were translated into English and transcribed verbatim, and the thematic analysis approach described by Braun and Clarke [29] was used. Overall, transcripts were coded in 3 steps: open coding, axial (thematic) coding, and selective inductive coding for understanding the experiences and expectation of informal caregivers. A mix of inductive and deductive approaches was used to determine the themes related to PU and IC needs. First, all the transcripts were read by 1 researcher (NS), and useful relevant fragments were selected and initially coded. Then, open codes were examined and arranged into themes. A second researcher (LMAB-J) coded 25% of the transcripts independently to validate the codes. The joint probability of agreement was 80%. The final categories were discussed by the 2 researchers until consensus was reached. This was done to deal with the researchers’ bias (if any). ATLAS.ti (ATLAS.ti Scientific Software) was used for these analyses [30].

### Meta-Inferences and Design Features

The understanding obtained from the survey and interview results was gleaned together in the form of meta-inferences by comparing, validating, and contrasting them, that is, more pragmatic interpretations. These meta-inferences were further used to elicit the design features for the interaction platform in accordance with the PSD model [21]. In general, the aim of the PSD model is to help designers or developers in developing solutions that facilitate behavior or attitude change. The intended interaction platform does not aim for behavior change in informal caregivers, but it requires persuasion to form or alter the behavior of informal caregivers for complying with the information communicated [31]. In this direction, the persuasion context and available categories of features in the PSD model appear to be advantageous and a step close to the design process [21]. Overall, 4 feature categories of the PSD model—primary task support, dialogue support, system credibility, and social support—were used here. Primary task support features can help caregivers in performing their primary task, that is, to organize and provide optimal care to the CR. Dialogue support features can help maintain a feedback loop and provide motivation for accomplishing the primary task of informal caregivers. System credibility features help designers in developing more credible and trustable systems. Finally, social support features help in intertwining the users with social communities in the same domain, thus supporting users through social influence. In the following sections, the obtained themes across this study were explored to observe their association with respective feature categories, if any.

## Results

### Participant Demographics and Care Elements

#### Survey

A total of 464 (6.69%) informal caregivers (mean age 58.3, SD 8.14 y) of older adults (mean age 86.7, SD 6.20 y) living alone were identified from the obtained 6934 responses. From the descriptions, it was observed that 76.9% (357/464) of the informal caregivers were women, and most informal caregivers (385/464, 82.9%) provide care to 1 older adult. A large group of informal caregivers were sons or daughters (360/464, 77.6%) of the CRs. While considering the high number of women as informal caregivers, it can be deduced that most of them were daughters of CRs. Finally, out of 464 informal caregivers, 119 (25.6%) responded for fall, 59 (12.7%) for nocturnal unrest, 81 (17.5%) for agitation, and all of them (n=464, 100%) responded for normal daily life. Table 2 provides an overview of the characteristics of the survey participants.

**Table 2:** Survey and interview participant demographics.

Demographics and care elements			Survey (N=464)		Interview (n=10)
Age of informal caregivers (y), mean (SD)			58.3 (8.14)		57.1 (6.45)
Age of CRs^a^ (y), mean (SD)			86.7 (6.20)		88.8 (6.01)
**Sex, n (%)**					
	Male	107 (23.1)		3 (30)	
	Female	357 (76.9)		7 (70)	
**Number of** **CR,** **n (%)**					
	1	385 (82.9)		10 (100)	
	≥2	79 (17)		0 (0)	
**Relationship with** **CR,** **n (%)**					
	Son or daughter	360 (77.6)		10 (100)	
	Son or daughter-in-law	46 (9.9)		0 (0)	
	Other family and friends	58 (12.5)		0 (0)	
**Participants or care scenarios, n (%)**					
	Fall	119 (25.6)		10 (100)	
	Nocturnal unrest	59 (12.7)		4 (40)	
	Agitation	81 (17.5)		3 (30)	
	Normal daily life	464 (100)		10 (100)	

^a^CR: care recipient.

#### Interviews

A total of 10 informal caregivers (mean age 57.1, SD 6.45 y) of older adults with cognitive impairment (mean age 88.8, SD 6.01 y) and living alone participated in the interviews. Among the 10 participants, 7 (70%) informal caregivers were women and 3 (30%) were men, and all of them (n=10, 100%) were either sons or daughters of the CR. All informal caregivers (10/10, 100%) had 1 CR. Of the 10 participants, all (n=10, 100%) answered for fall and normal daily life, whereas 4 (40%) answered for nocturnal unrest and 3 (30%) for agitation. Table 2 provides an overview of the characteristics of the interview participants.

### Experiences With Current Care Solutions

#### Theme 1: Experiences With Sensing Systems

From the interviews, it can be concluded that most informal caregivers (9/10, 90%) use multiple technological interventions in their current caregiving situations to assist them in delivering timely care to their loved ones. These sensing modalities include cameras (P6 and P10), voice-activated systems having microphones (P6), door alarms (P2 and P4), wearable fall alarms (P1, P4, P5, P3, P7, and P8), and device-free systems (P5 and P6).

##### Camera-Based Sensing System

The informal caregivers reported both positive and negative experiences with camera-based monitoring solutions. Although such solutions provide a precise picture of the situation in the house of the CR, they require constant attention from the informal caregivers:

Cameras really helps a lot, because she sometimes forgets [that her mother is not alive] and wants to go to see her mother. But when she is packing a bag and if we see that on camera, we can stop her.

P6

The informal caregivers must either monitor continuously or make a conscious effort to periodically check on the CR. This process is fatiguing and causes informal caregivers to worry about times when they are unable to observe the CR:

Usually, I am watching her more now-a-days [because of her illness] with the camera. But for example, I am not watching now, she could be doing anything right now and I have no idea. With such a system, she can actually monitored 24 hours a day.

P6

In addition, using cameras for monitoring requires line of sight, necessitating multiple cameras to cover different areas of the house:

The camera in the living room does not give notification. I can just watch continuously as long as she is line of sight.

P6

Informal caregivers also mentioned ethical and privacy concerns, especially when it comes to personal spaces such as bedrooms or bathrooms, and oppose their use in such areas.

##### Voice-Activated System

To get an insight into the real-time situation in the house of older adults with cognitive impairment, an informal caregiver deployed a voice-activated (microphone-embedded) system. The informal caregiver reported that microphones in Google Nest are very sensitive and can catch sounds from neighbors:

Google Nest is advantageous, because if she’s in the bedroom with the doors closed, I can still hear her if she makes some noise.

P6

Google nest is so sensitive that at times when she is turning the pages of a book near to the system, it seems as if an earthquake is going on or when you are in the kitchen, picking up a pan, then you think: the building is collapsing. That’s how much noise is there. At first it really frightened me.

P6

##### Alarm-Based Sensing System

Most informal caregivers (7/10, 70%) used an alarm system, which included either a door or wearable fall alarm. Informal caregivers found the door alarm to be highly practical, as it enables (known) visitors to enter the house without disturbing the CR and can also be activated in case of an emergency. In contrast, the wearable fall alarm, which can be activated in the event of a fall, was not frequently used. The reason for this is that CRs often dislike consistently wearing the alarm. Even if informal caregivers convince them to wear it during the day, CRs often remove it at night, increasing the risk of falls when they wake up in the night (eg, for toilet visits). Furthermore, cognitive impairment often causes CRs to forget to charge or replace the batteries or even forget to press the button during an incident. However, despite these disadvantages, informal caregivers take a leap of faith that the personal alarms will function in case of emergencies, providing them with a sense of security and reassurance:

Well, the personal alarm has one big disadvantage: my mother fell once, and she has one of those nice alarms around her neck, but she just forgets to press it.

P5

##### Device-Free Sensing System

Some of the informal caregivers (P5 and P6) were using device-free sensing systems to monitor the real-time situations in the house of the CR. An informal caregiver deployed a sensor in the corridor near the main entrance of the house to track the movements of the CR:

The sensor in the corridor gives a signal when there is any movement. The door to the toilet and the bathroom is also in the corridor, so for example, if I get a notification I see [with the help of camera] that she is going to the bathroom. I can also see if she walks to the front door then she usually goes outside but she doesn’t do that very much anymore.

P6

Another informal caregiver, who used a more advanced device-free sensing solution called Sensara highlighted its value in providing insights into the daily life activities of the CR. However, they also identified a major drawback, which was the lack of communication of logical and situation-aware information:

Yes, I think it’s a nice system but one shortcoming. I think that it should be actually linked to the agenda of care recipient. At the moment, when she’s not at home, for example, she has to walk outside for physiotherapy, she’s out for an hour. The system doesn’t take that into account. It only registers visitors coming in and visitors going out. And since my mother has no physical sensor on her body, the system registers and sends notifications that she has no movement during that time which is not true.

P5

#### Theme 2: Experience With IC

In the current health care infrastructure, the sharing of information between informal caregivers and formal caregivers is still a bit conventional, Such that, when informal caregivers want any specific information, they can try to contact formal caregivers or care organizations, where, if they are persistent with their request, they can get the answers. An informal caregiver said the following:

I am proactive. I intervene with organization if I see that things are really not going well. Every now and then I have a conversation with the management because they get stuck in their own rules or they fail because they just have too few resources/people. In these situations, I go to the management, until I have sorted things out for my mother.

P5

In contrast, advanced interaction platforms such as Caren exist to facilitate direct connections among CRs, formal caregivers, and informal caregivers. In general, various types of formal caregivers, such as district nurses and personal care assistants, are involved in providing care for older adults with cognitive impairment. According to informal caregivers, formal caregivers tend to primarily communicate and share information among themselves, with limited active communication directed toward informal caregivers. However, to keep informal caregivers informed, formal caregivers often provide brief written reports or updates. If more detailed information needs to be conveyed, informal caregivers are typically responsible for arranging it themselves, such as by making phone calls. Thus, informal caregivers feel a lack of active involvement from responsible care organizations and a lack of a comprehensive overview of the information flow in care platforms:

I must say that there is not a lot of communication. They communicate mainly with themselves [different formal caregivers], but they do note everything in the folder, so I can see that.

P2

If I write something in a message [one of the functionality in Caren platform], I don’t know if the home care can see those messages or they are only for other informal carers. But, it would be nice if I could communicate with home care company via platform. Because last week it was her birthday, we picked her up for the evening meal. Then we must call the organization responsible for food. And they don’t really have an emergency number or direct contact.

P6

Taking a step further, when modern sensing systems are leveraged in care, care platforms (web or mobile app) are developed specifically to communicate the gathered sensing information such as Sensara alarm. Through such systems, notification or reports can be sent to informal caregivers, enabling them to gain a comprehensive understanding of various daily life activities and emergencies:

I can estimate that [daily-life activities] quite well I can also see how often she goes to the toilet, how often she walks during the day, how often she walks around the room, how often she goes to the kitchen. I also see when she goes to bed and when she goes out at night and when she goes back, that’s all information that comes through fairly well.

P5

### Expectations From Future Care Solutions

#### Theme 1: Centralized Care Approach

Informal caregivers perceive the current care infrastructure as fragmented, characterized by the use of diverse communication channels among different care organizations. For instance, the Caren platform facilitates communication with daily care assistants, but contacting the care organization responsible for food planning can only be done by calling their customer support. Among all communication challenges, informal caregivers find it particularly challenging to establish connections with geriatricians or general practitioners owing to their limited time availability. Therefore, informal caregivers expect care solutions that integrate multiple formal caregivers or organizations into a unified platform to optimize their care planning. According to informal caregivers, a centralized approach to care can also enhance care insights for formal caregivers. A history of actions taken by different formal caregivers can help other caregivers (such as emergency services) to understand the situation and address problems simultaneously:

In my experience when I need the doctor in Boekelo, it is often difficult. If it is Friday, I can only go or call the doctor until three o’clock. Otherwise, I have to call the weekend service who might not have idea on my father’s health. So, if I could still get in touch with the GP or something via that platform to discuss something, it would be nice.

P3

Furthermore, informal caregivers also acknowledge that not all formal caregivers or informal caregivers require the same level of information. Therefore, they should be able to customize which information will be shared with which stakeholder in centralized care:

In care, you always have to make sure that you are sending the right message. There should also be a possibility that you only communicate with informal caregiver or formal, but we don]t have that. You still send the message, but now you should always be conscious that you are going to share the correct message [as it goes to everyone].

P8

#### Theme 2: Empathy in Care

In general, empathy is considered as a crucial aspect of our society as it enables individuals to understand and connect with others on a deep level. In the context of care of older adults with cognitive impairment, empathy becomes even more important. It involves recognizing and experiencing the emotions, experiences, and perspectives of older adults with cognitive impairment and responding with appropriate care and concern. Informal caregivers expect the entire care system, including modern care solutions and formal caregivers, to demonstrate more empathy toward older adults with cognitive impairment. According to them, it can foster better relationships between daily care providers and older adults with cognitive impairment:

That’s actually the empathy, that the staff have towards my mother. My mother has only one interest that is animals/pets and they all show pictures of their pets to her. But also, when they walk their dog, they come in for a second with the dog and to make her happy. They should definitely not reduce that.

P5

However, most informal caregivers felt the lack of emotional connection from formal caregivers toward older adults with cognitive impairment. This lack of connection may be attributed to the fact that, in many cases, a new care staff attends to the older adults with cognitive impairment, which in combination with cognitive impairment can lead to increased irritability or aggression toward the caregivers. Overall, informal caregivers suggest that formal caregivers should adopt empathic care approaches when interacting with older adults with cognitive impairment. They feel that offering a personal touch, such as discussing emotional matters, is necessary as it gives confidence to older adults with cognitive impairment and can provide them comfort—for example, engaging in conversation before starting their work and sitting next to them during meals as opposed to a more transactional or corporate care approach, where caregivers simply complete their tasks and leave:

It sounds a bit corporate, but home care organizations come, finish their tasks, and as soon as they’re done with that, they’re gone too. A better way to do it, for example on Sunday, when they come to provide her a meal. They don’t sit next to my mother and say: I have heated the food. It’s nice now. When are you going to start eating? They don’t do all this. There is some discontent there.

P9

#### Theme 3: Adaptable Sensing Solution

Across the interviews, a wide variation in care situations was observed. This divergence could be attributed to the progression of illness, comorbidities among CRs, and personal changes in the lives of older adults with cognitive impairment or their informal caregivers. For example, for most informal caregivers, fall emerged as a priority monitoring concern, whereas another informal caregiver (P7) reported that it was not a priority for them as their CR was bedridden and unable to move around in the house. Such evolving changes affect the overall care needs, necessitating adaptive sensing solutions that can adjust to evolving care needs.

Moreover, it was interesting to note that informal caregivers became more willing to accept diverse sensing solutions, including cameras, depending on the specific care situation. This willingness stemmed from their desire to ensure the safety of their loved ones, despite the ethical concerns associated with cameras or voice-activated devices. For example, an informal caregiver (P6) installed a camera in the living room to track the movements of the CR and a voice-activated device (having a microphone) for situations when the CR is out of sight from the cameras.

However, the use of voice-based coaching solutions in the care of older adults with cognitive impairment was found to be somewhat debatable based on the opinions expressed during the interviews. An informal caregiver emphasized that voice-based coaching could facilitate the eating and drinking habits of older adults with cognitive impairment, especially in cases where sensing systems are limited in their capabilities such as motivating the older adults with cognitive impairment to have warm meals or inquiring about their well-being if no movement was detected for an extended period among other functions:

What I do notice is that she eats less and less hot meals and more and more bread. So, she does eat, but not always well or healthy, but those are things you can’t force, and that’s what I find so difficult. The system can’t do that either, if system can pass a voice message, then someone would think: oh yes, I can make a warm meal or eat something warm instead of bread. So, you can guide someone in that. It would apply to certain people and not all.

P2

However, another informal caregiver expressed concerns about the potential confusion that voice-based instructions may cause for older adults with cognitive impairment who are not very alert:

Yes, we don’t really want to use the voice. Because she is demented and then suddenly she hears a voice that she doesn’t actually see, I have the idea that she will not comprehend it.

P6

Overall, it is evident that integrating multiple sensing modalities can be crucial in developing a comprehensive and adaptable care solution that addresses the emerging needs of both CRs and informal caregivers.

#### Theme 4: Trust and Reliability

Most informal caregivers showed trust in the care solutions. They widely use different types of technology to assist them in the care for older adults with cognitive impairment, which helps them to attain some level of peace of mind*:*

I trust technology, so I trust the system, if set up properly, the sensors will do their job and the data will be recorded and filtered into something that is useful to me. This useful information can give me peace of mind.

P5

However, to trust the system, they expect it to be reliable, such that it should not have several false alarms:

So, it must be reliable. There shouldn’t be false alarms every time that make you think: it’s not really that much use to me.

P9

#### Theme 5: Privacy and Security

An ethical dilemma regarding the choice between privacy and security was observed among informal caregivers of older adults with cognitive impairment. As the condition of cognitive impairment advances or comorbidities emerge, the demands for care increase, leading to the prioritization of safety or well-being of the CR over privacy. In such scenarios, they may advocate for the installation of obtrusive sensing devices such as cameras in the homes of their CRs. However, this does not imply that privacy is disregarded entirely but they place their trust in technology developers:

Yes, look, with camera privacy is gone. And yes, she just needs to be watched, and whether that is done with a USS or with a camera, or with someone being present, the privacy is just a lot less. And then you don’t put a camera in the bedroom because of privacy, yes, but you still want to keep an eye on her, because she can also fall in the bedroom.

P6

Privacy should be granted but it is secondary to security. But no one should be able to influence the system with false information [like hacking the system].

P5

### PU of USSs

Table 3 presents the results of both the survey and interviews. PU of USS measured through the Technology Acceptance Model PU construct was found to be positive across the informal caregivers (mean score 4.61, SD 1.32; 7-point scale). They believe that it provides reassurance and peace of mind owing to the 24/7 monitoring of CRs, thus reducing the time and effort invested in monitoring. With proper IC, informal caregivers can also prioritize and optimize their care plans, which can improve the quality of life and ensure timely care of older adults with cognitive impairment. Moreover, the long-term monitoring capabilities of USSs can provide insights into the onset of new illnesses, thereby helping formal caregivers to tailor care plans accordingly:

It would give me peace of mind. The quality of care can also be improved by using this system, because I would be able to provide more targeted care. I think that the same data is also important for the professional as they can also adjust their professional care much more specifically.

P5

With such a system, she can be actually monitored 24 hours a day.

P6

Furthermore, regarding usefulness for stakeholders, informal caregivers found USSs more supporting for themselves in facilitating their care task (mean score 3.72, SD 0.05; 5-point scale) compared with enabling independent living of their CR (mean score 3.27, SD 0.06; 5-point scale). In interviews, informal caregivers expressed that although USSs appear as a logical choice for them, older adults with cognitive impairment themselves may not perceive their value, as they may not acknowledge the necessity for care:

My mother herself would not be in favor because she does not see the problem. But yes, it [USSs] seems logical to me. Yes, I would be willing to use.

P2

They further added that these solutions are more appropriate for CRs whose motor functions are intact, rather than patients who are terminally ill, unless USS also provides insights into physiological activities:

I am one hundred percent convinced that the system has added value for people who have not yet reached the stage [terminally ill] and can still stand on their own feet in a safe and responsible manner. For example, it can keep a track of medication or food intake.

P7

Finally, regarding monitoring scenarios, informal caregivers found USS to be comparatively more useful for monitoring emergency scenarios (mean score 4.09, SD 0.04; 5-point scale) than monitoring daily life activities (mean score 3.50, SD 0.04; 5-point scale). This is because in case of emergencies, failing to take appropriate, timely actions could potentially worsen the CR’s condition:

Suppose he falls, and I am unable to visit him that day, and Livio [the care organization] also doesn’t come to shower him. In that case, there might be no one to attend to him. If I were to go there the next day, it is quite possible that he would be lying there all night or throughout the day. Such a situation is completely unacceptable.

P3

**Table 3:** Perceived acceptance of USS obtained using the mixed methods approach^a^.

		Survey score, mean (SD)
Perceived usefulness (TAM^b^)^c^		4.61 (1.32)
**For stakeholders**		
	Support for informal caregivers^d^	3.72 (0.05)
	Independent living of older adults with cognitive impairment^d^	3.27 (0.06)
**Care scenarios**		
	Daily life activities^d^	3.50 (0.04)
	Emergency^d^	4.09 (0.04)

^a^Findings from interviews—reassurance and peace of mind, 24/7 monitoring, prioritization and optimization of care plans, and more useful during emergencies.

^b^TAM: Technology Acceptance Model.

^c^Question from TAM scale, measured using a 7-point scale.

^d^Customized question, measured using a 5-point scale.

### IC Needs and Requirements

#### Overview

The IC needs of the informal caregivers of the older adults with cognitive impairment living alone were gathered by using both survey and interviews for 4 care scenarios including fall, nocturnal unrest, agitation, and normal daily life. These needs are presented in the 6 themes: mode of IC, content of the communicated information, timing of IC, intended users for IC, feedback to the system for self-learning, and dialogue support needed to users. Finally, meta-inferences based on the survey and interview results were deduced.

#### Theme 1: Mode of IC

The mode of IC varies with respect to type of situation. The multiple response set indicated that most informal caregivers (61/152, 40.1%) wanted to receive emergency (such as fall) information immediately via call. In contrast, for the situations that are urgent but not emergency such as nocturnal unrest and agitation, they preferred either self-check on the platform (26/68, 38% and 42/112, 37.5%, respectively) or a notification on time indicated by them (25/68, 37% and 40/112, 35.7%, respectively). However, for normal daily life updates, they prefer to self-check when they have time (267/464, 57.6%).

The interviews were found to be consistent with the survey results. In emergency situations, informal caregivers preferred to receive immediate calls or notifications to ensure timely response, whereas for urgent scenarios, they preferred self-check options as it allows them to check whenever they have time. When considering agitation scenario, an informal caregiver said the following:

If system indicates agitation, would I go to my mother for that? No, because that can wait, and I’ll go tonight and then check it.

P7

Regarding normal daily life, another informal caregiver said the following:

If I don’t get a message, then I assume that it’s going well. Otherwise, you get lots of messages.

P3

#### Theme 2: Content of the Communicated Information

##### Notification

Interestingly, most informal caregivers prefer to receive the information about emergency in either raw (48/115, 41.7%) or interpreted (48/115, 41.7%) manner, whereas they preferred raw data (24/59, 41%) as content of IC in nocturnal unrest situation. Their preference changes for agitation and normal daily life notification—they wanted interpreted data with suggestion (27/81, 33% and 147/464, 31.7%, respectively). On the basis of interviews, it can be observed that, along with care scenario, each informal caregiver has their own preference for the content of the notification. In the context of fall situation, an informal caregiver said the following:

A quick notification that something is wrong, something short. If I need to know more about this, I can click further.

P6

It was also observed that some informal caregivers were unaware of the significance of interpreted data; after explaining, they were inclined toward getting notification in interpreted way*:*

I hadn’t really looked at it this way yet. Interpreted data can be important, especially with a heart attack, then I know, I must call in other help, I must act quickly.

P2

##### Reports

For situations requiring long-term monitoring such as nocturnal unrest (21/59, 36%) and agitation (28/81, 35%), informal caregivers prefer the system to observe for a few days and then share a report to them and formal caregivers. This is because scenarios such as nocturnal unrest can be experienced owing to some personal circumstances and thus should be measured for a bit longer period before sending. Regarding nocturnal unrest, an informal caregiver said the following:

Suppose the system signals that she has been sleeping more restlessly for two weeks than the entire period before. And if I know her friend died recently, then I think it can be explained, but is then the system can be made aware by caregivers that there is no need to monitor more carefully.

P4

Regarding agitation scenario, another informal caregiver added the following:

It is good to know if something occurs structurally. If it happens every night for a week, then of course you want to make sure that there are indeed precautions. And if you notice she, does it once every three nights and after three weeks it’s over. Then you don’t want any extra solutions. So, if you get this report, you can make decisions based on that information than your own intuition.

P9

##### Confidence Prediction

It was observed that sharing the confidence percentage of system’s predictions might be more informative rather than just providing binary outputs:

With 20 percent [prediction confidence percent] you worry a little less. But that doesn’t say everything. Then, yes, the system hasn’t seen it well enough, but at least you can take action. You can call and say: “Oh, is it true that you have fallen?”

P2

That does increase confidence. Then I understand that it is very difficult for system to notice her eating behavior. So, then I can always call her and ask: what did you eat? It is nice indication and then I can still determine what happened that way.

P4

Some informal caregivers also indicated that knowing the confidence in prediction will not change their level of concern and they will still call and ask if their CR is doing well.

#### Theme 3: Timing of IC

According to the survey, most informal caregivers wanted to receive emergency (such as fall) information immediately (58/115, 50.4%) or within 5 minutes of the incident (51/115, 44.3%). The immediate notifications were found to be valuable as it can help informal caregivers in providing on-time care:

I would like to be notified immediately [about fall] because then I can react and ask how it’s going, even if she gets up straight away.

P2

Regarding the nocturnal unrest scenario, most informal caregivers (33/59, 56%) wanted to personalize when and if they want to receive the alert about whether the CR is in and out of the bed. According to them, nocturnal unrest is not an emergency scenario, but having detailed information about it is also important to gain better insights into the CR’s health:

Next morning, I would like to know, if she is slept, when she wakes up, when and how much she sleeps, and what does she do if she is awake?

P1

Moreover, most informal caregivers indicated that they can receive alert about agitation and normal daily life at any point of time (52/81, 64% and 295/464, 63.6%, respectively). However, contrasting opinions were found in interviews. Informal caregivers express a preference for not receiving agitation information during nighttime, but rather desire it to be personalized (similar to nocturnal unrest):

Well, when it comes to sending notification in night, it will be at the expense of my own night’s sleep unless there is actually no life-threatening situation so I would like to personalize that [the time of receiving alert].

P9

Regarding the timing of information sharing in normal daily life scenarios, mixed opinions were identified among informal caregivers. Although some caregivers value receiving positive information at the end of the day, others believe that such information is unnecessary and could contribute to information overload. Consequently, they prefer to look at information at a more convenient time by themselves:

It is always good to receive something when things are going well, and not only when things are not going well, so that is a bit of reassurance and I appreciate that. The notification can be sent at the end of the day.

P1

I will look that up myself when it suits me.

P5

#### Theme 4: Intended User for IC

A preference about the stakeholder to whom information can be communicated was observed. Survey suggested that most informal caregivers (44/115, 38.3%) want the system to alert them along with formal caregivers. This is because formal caregivers can ensure medical care if needed. An informal caregiver stated the following:

You must ask yourself: what is the contribution that someone can make to the problem, and who has priority? Is it important that I know that my mother is lying there with a broken leg or first a doctor who can offer real help knows. I do think you should inform me, but not first.

P7

Moreover, in nocturnal unrest and agitation scenario, they expect the system to directly contact formal caregivers (21/59, 36% and 39/81, 48%, respectively). Informal caregivers believe that formal caregivers are usually available at night and can act promptly, and thus, they should be alerted first:

The first, formal caregivers should be informed because they are on call and if necessary, home care will decide to call [inform] family.

P10

Finally, in normal daily life scenario, informal caregivers did not expect the system to communicate with any stakeholder. They strongly insisted to personalize who can see this information:

Well, you should be able to set that up with your own group of family caregivers so that they see that, but the GP doesn’t need to see that, I think.

P6

#### Theme 5: Feedback to the System for Self-Learning

Most informal caregivers (in both survey and interviews) indicated their willingness to provide feedback to the system based on its predictions. Specifically, 59.1% (68/115) of the informal caregivers who responded for fall, 64% (38/59) of the informal caregivers who responded for nocturnal unrest, 25% (20/81) of the informal caregivers who responded for agitation, and 42.2.% (196/464) of the informal caregivers who responded for normal daily life were willing to give feedback to the system with an aim to improve the system’s predictions in the future.

In the interviews, all the informal caregivers (10/10, 100%) were positive about giving feedback to the system to improve it for future users. Informal caregivers said the following:

Well, it would be short and sweet, but I would do it.

P2

Yes, I would also like to help improve the system.

P6

Yes, it’s good to improve the system again for future users.

P10

Furthermore, an informal caregiver further explained and understood the need of such feedback for the improvement of such novel technology. The informal caregiver added the following:

That’s the most important thing, you really need feedback, and certainly in the initial phase. I always say like this: hey, it starts with chaos and then you go to structure. And when you put structure into practice, you need feedback on it. Today there are enough digital possibilities for getting the feedback to make the system reliable and that is very important.

P7

#### Theme 6: Dialogue Support (Compliments and Suggestions)

A mixed response from informal caregivers in survey about receiving compliment from the system was observed based on care scenario at hand. Most of those who responded for fall (48/115, 41.7%), agitation (31/81, 38%), and normal daily life (147/464, 31.7%) were not sure if they want to receive a compliment. In contrast, most of those who responded for nocturnal unrest (21/59, 36%) were willing to receive compliments for their care tasks. The interviews partially supported the survey results. It was observed that if the compliment is followed by some suggestion to coach informal caregivers based on their actions, it appears more logical to informal caregivers:

I am always open to advice. Compliments are always nice too.

P2

Well, I think a compliment without explanation can be debatable. I think and completely agree that we should get compliments also in a normal daily situation. Like we want to underline the things that are not going well in feedback, but things that are going well, you should certainly also be acknowledged. So, in that respect I am in favor of it.

P7

### Design Requirements

Meta-inferences were drawn by pragmatically comparing and contrasting the IC needs obtained from the survey and interviews (Table 4). Furthermore, these meta-inferences were used to elicit design features in accordance with the PSD model. Table 4 illustrates the identified PSD features among 4 feature categories (primary task support, dialogue support, system credibility, and social support) by the authors to facilitate the designing of the interaction platform.

In the primary task support category, 3 features, namely, *reduction, tailoring*, and *personalization* were identified as useful in supporting informal caregivers in caregiving via the interaction platform. The *reduction* feature facilitates the breakdown of complex tasks into small ones, such as calling or notifying informal caregivers in the event of an emergency or urgent scenario, without requiring them to access the platform. The *tailoring* feature enables the platform to cater to different stakeholder groups, such as formal and informal caregivers, by tailoring the content as per their needs. Finally, the *personalization* feature appeared to be very crucial in developing such an interaction platform, as every care situation is different and results in diverse care needs. Personalization is required to account for differences in informal caregiver preferences, followed by the updating of care needs owing to disease progression or emerging comorbidities. These features can be found useful irrespective of care scenarios.

In the dialogue support category, 2 features, *reminder* and *suggestion*, were used to enhance the interaction between informal caregivers and the interaction platform specifically for emergency scenarios. *Reminders* can be sent to informal caregivers in case they fail to respond to emergency calls or notifications. In addition to notifications, *suggestions* can be sent to aid informal caregivers in dealing with emergency or urgent scenarios. When faced with emergency situations, informal caregivers may become overwhelmed and unable to think or act quickly. Suggestions with actionable steps can assist in providing the appropriate care to the CR.

Finally, regarding system credibility support and social support, 2 features, *trustworthiness* and *social learning*, were recognized to make the platform persuasive in general. To establish *trustworthiness*, the interaction platform is expected to send reliable, unbiased, and fair information to informal caregivers. For example, the system’s confidence percentage in prediction can be shared through notifications to maintain transparency. *Social learning* can benefit informal caregivers of older adults with cognitive impairment in multiple ways. It can facilitate connections with other caregivers for sharing knowledge, skills, and strategies to provide care in an efficient way. Furthermore, caring for older adults with cognitive impairment can be emotionally challenging, and social communities, if they exist, can provide empathetic support and help develop effective coping strategies for managing stress, anxiety, and other negative emotions that may arise during caregiving.

**Table 4:** Requirements (meta-inferences) drawn from the obtained information communication (IC) needs and persuasive system design (PSD) features.

Theme	Requirements (meta-inference)	Category-wise PSD features
Mode of IC	Preferences for the mode of IC were found to vary based on the severity of scenarios. Emergencies, such as falls, require a *call or alarm notification* to immediately alert informal caregivers. *Reminders* may be sent if needed, if the informal caregiver is not responding to the sent notification. Scenarios that require awareness of informal caregiver but not immediate attention, such as nocturnal unrest or agitation, were preferred to be *communicated via direct notification*. However, no communication is necessary for scenarios where everything is going well, and informal caregivers may self-check the updates at their convenience.	Primary task support Reduction Dialogue support Reminders
Content of the communicated information	The content of notifications was discovered to be more influenced *by individual preferences* than by the care scenario. Certain informal caregivers favored receiving raw information, whereas others preferred interpreted information with suggestions. However, for immediate situational comprehension, informal caregivers generally preferred short and concise information, such as raw data. After being informed about the significance of interpreted data, they recognized its potential. For long-term monitoring scenarios such as nocturnal unrest or agitation, detailed reports were deemed useful for analyzing, revisiting, and concluding the care needs, for both informal caregiver and formal caregivers. Thus, *these reports can be adapted depending on the stakeholder (formal caregiver* *or* *informal caregiver).*	Primary task support Personalization Tailoring
Timing of IC	Time of sending the notification varies across scenarios. During emergencies, where the life of the older adult with cognitive impairment is at risk, information should be *communicated immediately (without requiring effort of log-in from the involved stakeholders)*. For other significant scenarios, which do not constitute an emergency, informal caregivers can *personalize the notification’s timing*. Monitoring CR’s^a^ daily well-being is reassuring, but informal caregivers may self-monitor on the platform. Informal caregivers intended to seek updates on daily activities such as eating, drinking, walking, and medication intake, but receiving notifications for each activity may be overwhelming unless requested otherwise based on their individual circumstances.	Primary task support Reduction Personalization
Intended users for IC	*Informal caregiversdesired to keepformal caregiversinformed, albeit on varying levels*. During emergencies, they preferred immediate system contact with formal caregivers as they can provide the necessary medical care. If the primary informal caregiver is unresponsive during an emergency, other informal caregivers can be contacted. Long-term monitoring reports about nocturnal unrest and agitation can be shared with formal caregivers to offer insight into disease emergence or progression, allowing for timely diagnosis. Similarly, informal caregivers wished to store normal daily life well-being information to draw insights together with formal caregivers if needed.	Primary task support Tailoring
Feedback to the system for self-learning	Informal caregivers were willing to provide feedback to the system based on its predictions to improve the trust of future users in the system. It was interesting to note that although *informal caregiver* *desires a trustable system*, they understand the need for feedback to make the technology more robust.	System credibility Trustworthiness
Dialogue support needed for users	A mixed response about providing compliments to informal caregivers for their care task was observed. However, if the compliments can be combined with *suggestions from experts* or *other (in)formal caregivers*, they are seen as more meaningful.	Dialogue support Suggestion Social support Social learning

^a^CR: care recipient.

## Discussion

This study offers insights into the development and use of sensor-based care solutions for the care of older adults with cognitive impairment from the perspective of informal caregivers. The findings from the study are discussed in 5 main aspects: experiences with diverse sensing solutions, expectations toward care infrastructure and sensing solutions; PU of USSs; varying IC needs; and use of PSD features.

### Experiences With Diverse Sensing Solutions

This study provided an overview of the different sensing technologies (such as cameras, wearables, and device-free sensors) currently being used by informal caregivers in care. Previous studies also indicated the need for technological interventions in in-home care [5,32]; however, they also highlight the disadvantages of obtrusive solutions such as wearables and cameras [17], despite that informal caregivers were found leveraging these solutions. In this study also, the participants reported some limitations of these sensing modalities, such as the need to be in the line of sight, privacy concerns, requirement to wear them 24/7, and lack of logical communication, but they also highlighted several benefits such as accurate and instantaneous monitoring, outdoor tracking, unobtrusive monitoring, and comprehensive understanding of the daily activities of the CR from them. Although these outcomes indicate the lack of solutions adhering to all the needs of informal caregivers, they also highlight the possibility that various sensing modalities can be advantageous in different applications. For instance, device-free solutions may be a suitable choice for in-home care [1], whereas wearables are more useful for outdoor tracking of older adults with cognitive impairment [33]. Nonetheless, to achieve a complete care solution, multiple sensing modalities might need to be integrated.

### Expectations Toward Care Infrastructure and Sensing Solution

To facilitate the future development of sensor-based care solutions, the study highlighted the expectation of the informal caregivers from in-general care infrastructure and sensing solutions. The existing care infrastructure in the care of older adults with cognitive impairment was also found to be fragmented. Specifically, the care information about older adults with cognitive impairment was found to be scattered at different avenues, a lack of coordination between the different stakeholders was evident, and informal caregivers experienced limited access to care services. In general, the fragmentation of care is a global problem that has been persistent in the care infrastructure for a long time [34], and so far, no concrete solution has been devised. Certainly, centralizing care infrastructures offers potential benefits in terms of reducing overall care costs, particularly in administration; improving the coordination of care services; and facilitating knowledge transfer among different organizations [35,36]. However, it is important to acknowledge the associated challenges, such as the initial setup efforts, potential perceived loss of control at the local level, potential increases in government expenses, and different policies at the national or organizational level [35-37].

Furthermore, a lack of empathy in care, specifically from formal caregivers, was observed in this study. Informal caregivers believe that taking an empathetic care approach will not only foster a relationship between the CR and formal caregiver but will also have positive and calming effects on older adults with cognitive impairment and hence should be integrated into care. According to the review by Moudatsou et al [38], several factors influence the development of empathy among care workers. These factors include a high number of care receivers, insufficient time, fear of violating professional boundaries, and a lack of education regarding empathy. To foster the integration of more empathetic practices, it is important to address these inhibiting factors during the training of formal caregivers and in their workplace environments [39].

Moreover, care situations are found to be dynamic; they are largely influenced by the progression of illness in the CR, emergence of comorbidities of the CR, and personal situations or preferences of both the CR and informal caregiver’s. Traditionally, informal caregivers are expected to become a part of the care team and are viewed as a conduit for information between care professionals and extended family members [40,41]. These caregivers are also responsible for managing their own physical, mental, social, and financial well-being. Therefore, any changes in their personal lives directly affect their ability to be present and provide care to their CRs. Furthermore, owing to the advanced age of CRs, they are more susceptible to chronic diseases, necessitating timely and meticulous supervision of care plans [42]. Overall, given these unpredictable situations, adaptable care solutions that take the specific needs of the CR and involved informal caregiver into account are required.

Most informal caregivers expressed trust in sensor-based care solutions, citing their reliance on technology to assist them in the care of older adults with cognitive impairment. A similar observation was made in a study with older adults, that is, older adults also emphasize the importance of reliability, as false alarms can undermine their trust in the system [43]. Informal caregivers recognize the trade-off between privacy and safety and express the need to strike a balance. They understand that installing cameras or other sensing devices may compromise privacy, but they see it as a necessary measure to ensure the well-being of their CRs, particularly as cognitive impairment advances or comorbidities emerge [44,45]. In addition, from the perspective of older adults, the use of ambient technological solutions was perceived as beneficial, as it can increase the sense of security among older adults who prefer to live alone in their houses [5,43]. Overall, the concerns regarding trust, reliability, privacy, and security are far more complex for informal caregivers than they appear; therefore, other responsible stakeholders such as designers, developers, and researchers should take these factors into account while designing or developing technology for the care of older adults with cognitive impairment.

### PU of USSs

The positive attitude of informal caregivers toward PU of USS appears to be encouraging for the development of such solutions. Interestingly, informal caregivers perceived USSs as more useful in assisting them in comparison with facilitating the independent living of older adults with cognitive impairment in their own homes, specifically in emergency scenarios. Upon successful implementation, USSs might reduce the care time needed from caregivers by monitoring older adults with cognitive impairment 24/7 [46]. Nevertheless, their response was still on a positive spectrum, indicating that if the system appeared to be useful after implementation for emergencies, the chances are high that they would accept it for monitoring daily life scenarios also. Existing literature also indicates the usefulness of USSs among other stakeholders including formal caregivers and older adults with cognitive impairment depending on care situations [3]. Interestingly, USSs can be used not only to cope up with the shortage of experienced formal caregivers but also to support formal caregivers with low education levels or caregiving experiences [47].

### Varying IC Needs

The findings suggest that IC needs vary depending on care scenarios and individual preferences. Specifically, IC needs were found to be consistent across emergency scenarios (falls) and normal daily life scenarios, with most informal caregivers having similar preferences. However, for short-term or long-term monitoring scenarios, such as nocturnal unrest and agitation, preferences varied and contradicted among informal caregivers, thus highlighting the importance of providing flexible communication options that cater to different levels of urgency and individual preferences. In general, the IC needs of informal caregivers of older adults is an understudied topic, but these findings resonate with the situation-dependent varying information needs of other care-requiring avenues such as caring for patients with cancer [48,49]. Therefore, the development of solutions that intend to communicate the information to caregivers might benefit from using user-centered design approaches, such as Centre for eHealth Research Roadmap, where at first, user needs are studied and prototypes are cocreated with end users [50].

### Use of the PSD Model

The use of the PSD model to elicit design features can help future design works in making informed choices [21]. Although the aim of this interaction platform is not to change a caregiver’s behavior or attitude, it focuses on establishing a trusted link between informal caregivers and the system itself, so that informal caregivers comply with the system when needed [30]. On the basis of the findings, it can be implied that a “one size fits all” approach for designing an interaction platform is not suitable [51,52]. Design features that facilitate personalized communication such as tailoring and personalization are strongly recommended. For example, the tailoring feature appears to be useful in catering to different stakeholders, whereas the personalization feature accommodates the individual needs of users within the same or different stakeholder group [53,54]. However, the level of personalization remains as a topic of ongoing debate, given the delicate balance between personalized experiences and potential intrusions on privacy [55,56]. Features such as reminders and suggestions were found to be particularly useful in emergency situations, whereas features such as reduction, trustworthiness, and social learning were identified as in-general design considerations for an interaction platform. Overall, the intended use (advantages and disadvantages) of PSD features should be considered carefully before their implementation [53].

### Strengths and Limitations

This study is a first of its kind, which provides a comprehensive understanding of the various perspectives (such as experiences, expectations, and usefulness) of the informal caregivers toward the development and implementation of USS. The early knowledge about factors that are critical for development can strengthen the future implementation of these advanced technologies. The study also adds to the limited literature about IC needs of informal caregivers. Another major strength of this study lies in the methodology used. The complementary use of survey and interviews helped in deriving informative results.

Furthermore, some limitations to this study can also be identified. First, most of the informal caregivers indicated the presence of cognitive impairment through their experience with the CR, but they might not have been formally diagnosed. This is a prevalent challenge in the realm of older adult care, as timely formal diagnosis of dementia is often elusive. In such cases, informal caregiver’s opinions are usually considered in the literature [24,25], and they hold substantial value in this study. Although this can be seen as a limitation in the context of older adults with cognitive impairment, the findings possess a high potential for generalizability in the broad realm of older adult care. Second, this study was conducted in the Dutch context, limiting the generalizability of results to other nations’ health care infrastructures, as different countries have different models and approaches to health care delivery and financing, influenced by factors such as cultural norms, political systems, and economic considerations.

Third, it is important to note that our study focused solely on the perspective of informal caregivers and did not include the perspectives of other directly associated stakeholders, such as formal caregivers (eg, case managers and general practitioners) or CRs. Considering the diverse roles of various stakeholders in the care of older adults with cognitive impairment, it is crucial to identify and involve them from the early stages of system development to ensure successful implementation [50,54].

Fourth, a lack of knowledge about advanced solutions such as USSs among survey respondents can be imagined. Although during interviews, comprehensive explanation was provided (both written and oral) to ensure participant satisfaction, the potential lack of understanding about the survey may have led to optimistic responses, particularly regarding PU. Therefore, future studies examining such solutions should consider educating participants beforehand to ensure a more informed and unbiased assessment of their opinions. Finally, it is worth noting that the number of informal caregivers interviewed in our study was relatively small compared with the survey participants. Although saturation was reached for responses related to normal daily life and the fall scenario, contradictory responses were obtained for the scenarios involving nocturnal unrest and agitation. However, these contradictory responses can be attributed to the need for personalization.

### Conclusions

In conclusion, the study empowers the future development and implementation of such advanced solutions by highlighting the experiences, expectations, needs, and requirements from the perspective of informal caregivers. The findings highlight the possibility of merging diverse sensing modalities, including wearables, cameras, and device-free sensors, to develop a more inclusive care solution for the care of older adults with cognitive impairment based on the care needs of the informal caregivers. Furthermore, informal caregivers expect the care infrastructure to adopt a centralized and empathetic care approach, with care solutions that are more adaptable, trustable, and privacy aware. They showed trust in sensor-based care solutions but also emphasized the importance of reliability and striking a balance between privacy and safety.

PU of USSs was on a positive spectrum, particularly for emergencies, indicating its potential in optimizing care plans for caregivers. The preferences for IC needs varied depending on the care scenarios at hand and individual preferences, thus mandating the involvement of concerned stakeholders in the development (ie, iterative user testing) and implementation stages (ie, selecting the right settings together with other stakeholders). The use of the PSD model resulted in various useful PSD features, which can be used in future studies aiming to design such platforms.

## Appendix

Multimedia Appendix 1 The survey questionnaire used for quantitative data collection.

Multimedia Appendix 2 The interview script used for qualitative data collection.

## Abbreviations

CR: care recipient
CSI: Channel State Information
IC: information communication
PSD: persuasive system design
PU: perceived usefulness
USS: unobtrusive sensing solution
