# Supplementary material for: Exploring the Needs and Requirements of Informal Caregivers of Older Adults With Cognitive Impairment From Sensor-Based Care Solutions: Multimethod Study
Source: JMIR Aging. 2023 Oct 25;6:e49319. doi: 10.2196/49319 (PMC10632915; doi:10.2196/49319)
Supplement: Multimedia Appendix 1 [file aging_v6i1e49319_app1.docx]

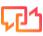
 [**Translated from Dutch to English - www.onlinedoctranslator.com**](https://www.onlinedoctranslator.com/en/?utm_source=onlinedoctranslator&utm_medium=pdf&utm_campaign=attribution)

**Appendix 1: Survey questions for informal caregivers**

**I am a carer**

**37.How many people do you provide informal care to? Please answer with numbers***


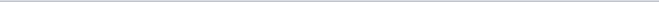


**38.How long have you been active as a carer?***

***Mark only one oval.***


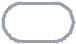
 **Less than 1 year**


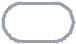
 **Between 1 and 3 years**


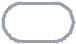
 **More than 3 years**

**39.How long have you been using Caren?***

***Mark only one oval.***


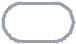
 **Less than 1 year**


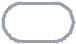
 **Between 1 and 3 years**


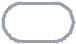
 **More than 3 years**

**40.Are you satisfied with Caren?***

***Mark only one oval.***


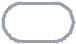
 **Very satisfied**


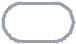
 **Satisfied**


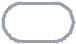
 **Neutral**


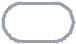
 **Dissatisfied**


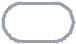
 **Very dissatisfied**

**https://docs.google.com/forms/d/18Pt5mtN4vZZkLIT3Xir3FLLfwuD5lzI3PSXlo3DSzIQ/edit** **13/38**

**25/04/2022, 11:49** **Questionnaire**

**When answering the following questions, think of one specific person to whom you provide informal care**

**41.What is the age of the person you provide care to? Please answer with numbers***


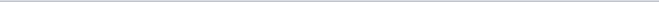


**42.How many healthcare organizations are you linked to in Caren?***

***Mark only one oval.***


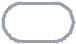
 **1**


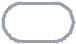
 **2**

**3**


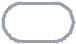
 **More than 3**

**43.Which healthcare providers are linked to you in Caren? Choose all options that apply to you***

***Check all that apply.***


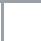

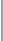
 **Practitioner (such as occupational therapist, physiotherapist, social worker, physician psychologist)**


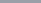


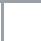

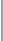
 **(district) nurse/nurse**


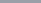


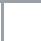

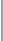
 **Caregiver**


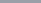

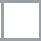


**Accompanist**


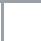

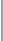
 **Does not apply**


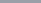


**https://docs.google.com/forms/d/18Pt5mtN4vZZkLIT3Xir3FLLfwuD5lzI3PSXlo3DSzIQ/edit** **14/38**

**25/04/2022, 11:49** **Questionnaire**

**44.How often do you visit the person you care for?***

***Mark only one oval.***


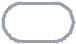
 **Several times a day**


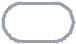
 **Once a day**


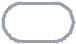
 **4-6 times a week**


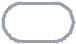
 **1-3 times a week**


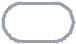
 **Less than once a week**


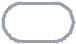
 **Monthly**

**45.What is your reason for providing care? You can choose multiple options***

***Check all that apply.***


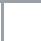

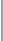
 **Old age**


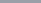


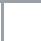

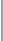
 **Dementia or mild cognitive impairment**


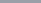


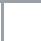

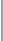
 **Physical disability**


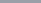


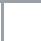

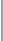
 **Mental disorder**


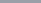


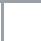

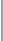
 **Visually or hearing impaired**


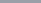


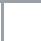

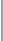
 **Intellectual disability Other:**


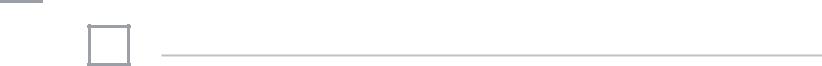


**46.How long have you been providing care to this person?***

***Mark only one oval.***


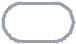
 **0 to 6 months**


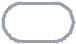
 **6 months to 1 year**


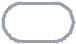
 **1 to 2 years**

**2 to 5 years**


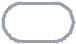
 **More than 5 years**

**https://docs.google.com/forms/d/18Pt5mtN4vZZkLIT3Xir3FLLfwuD5lzI3PSXlo3DSzIQ/edit** **15/38**

**25/04/2022, 11:49** **Questionnaire**

**47.What is your relationship to the person you care for? I am***

***Mark only one oval.***


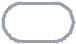
 **Husband/Partner**


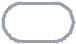
 **Daughter son**


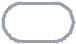
 **Daughter-in-law/son-in-law**


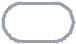
 **Granddaughter / grandson**


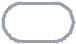
 **neighbors / friend**


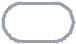
 **Other:**


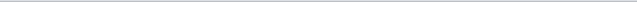


**48.Which living situation applies to you?***

***Mark only one oval.***


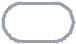
 **I live in the same house as the person I care for** ***Skip to question 50***


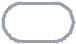
 **The person I care for does not live in my house** ***Skip to question 49***

**Doesn't live in my house**

**49.How far away do you live from the person you care for?***

***Mark only one oval.***


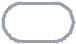
 **I live very close by (less than 5 minutes travel time)**


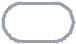
 **I live about 15 minutes away**


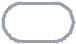
 **I live about 30 minutes away**


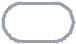
 **I live about 1 hour away**


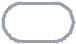
 **I live more than an hour away from home**

***Skip to question 50***


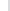

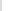


**https://docs.google.com/forms/d/18Pt5mtN4v ZZkLIT3Xir3FLLfwuD5lzI3PSXlo3DSzIQ/edit** **16/38**


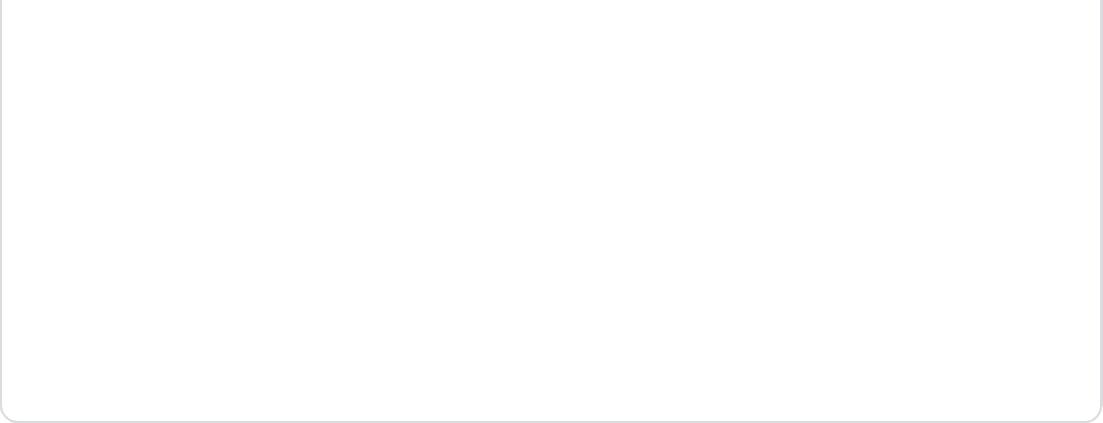
**25/04/2022, 11:49** **Questionnaire**

**sensor system**

**Smart box in the house**

We are researching on a new unobtrusive sensing technology to recognize various care situations in older adult care. It is unobtrusive, which means that your loved one does not have to wear anything on the body and does not require line of sight to be able to use this device. As you can see in the image, a small sensing box, can be placed in the corner of the house. This smart box can track various activities and observe important changes, such as drinking or eating less, change in heart rate or breathing, nocturnal unrest, but also emergencies such as a fall. The system can learn to recognize certain situations and inform you as a caregiver about them. In order to develop this system, we therefore want to understand what your needs are and requirements from such as system. Specifically, we want to ask you which situations are most important to you? when would you like to receive information from the platform? what exactly do you want to know? how and when you want to know. Keep this smart box in mind when answering the following questions:


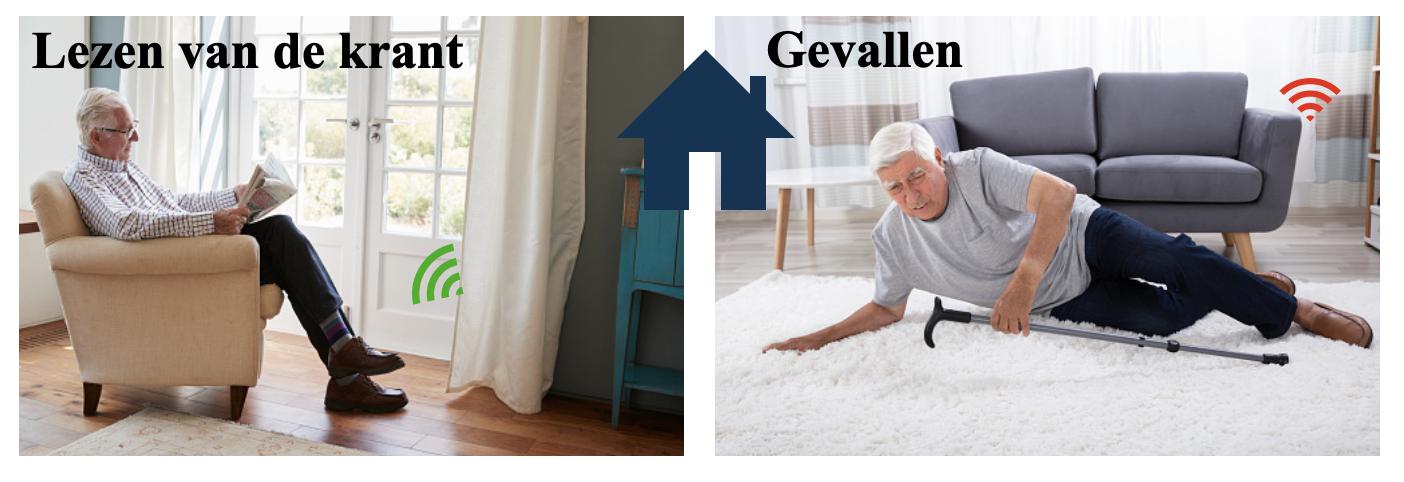


**50.Would you accept this smart box in your loved one's home to monitor their daily activities such as eating, drinking and taking medicines?***

***Mark only one oval.***


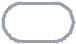
 **Not at all**


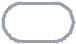
 **Not**


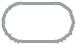
 **Maybe**


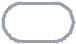
 **Well**


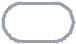
 **Certainly**

**https://docs.google.com/forms/d/18Pt5mtN4vZZkLIT3Xir3FLLfwuD5lzI3PSXlo3DSzIQ/edit** **17/38**

**25/04/2022, 11:49** **Questionnaire**

**51.Would you accept this smart box in your loved one's home to monitor emergency situations such as a fall?***

***Mark only one oval.***


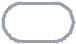
 **Not at all**


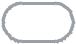
 **Not**


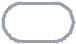
 **Maybe**


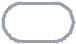
 **Well**


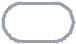
 **Certainly**

**52.Do you think that this smart box can support you in providing better care and care when it is desired?***

***Mark only one oval.***


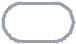
 **Not at all**


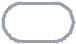
 **Not**


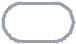
 **Maybe**


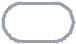
 **Well**


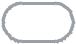
 **Certainly**

**53.Do you think this smart box will help the person you care for to live independently and safely at home for longer?***

***Mark only one oval.***


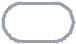
 **Not at all**


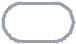
 **Not**


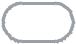
 **Maybe**


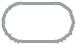
 **Well**


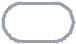
 **Certainly**

**https://docs.google.com/forms/d/18Pt5mtN4vZZkLIT3Xir3FLLfwuD5lzI3PSXlo3DSzIQ/edit** **18/38**

**25/04/2022, 11:49** **Questionnaire**

**54.Would you like to use such a smart box in the home of the person you care for?***

***Mark only one oval.***


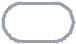
 **Certainly**


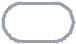
 **Probably**


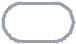
 **Maybe**


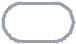
 **Probably not**

**Certainly not**

**Information platform**

**After the smart box has collected information from the person you care for, it is of course important that this information reaches you in the right way, via a platform. This information helps you plan visits, reassure you when everything is in order, or alert you in case of an emergency. We would like to understand what information is important to you.**

**Information platform with smart locker**

**https://docs.google.com/forms/d/18Pt5mtN4vZZkLIT3Xir3FLLfwuD5lzI3PSXlo3DSzIQ/edit** **19/38**

**25/04/2022, 11:49** **Questionnaire**

**55.What information would you like to receive? (multiple answers possible)***

***Check all that apply.***

**All information that can be collected Only information**

**about emergency situations (such as a fall) Information**

**about sleep quality/night restlessness Information about**

**medication intake**

**Information about water and food intake**

**Information about personal hygiene (such as washing, brushing teeth and dressing). Information about daytime restlessness/agitation**

**I do not want to receive this information**

**56.On which device would you like to receive information about the smart box?***

***Check all that apply.***

**Laptop/computer**

**Tablet**

**Telephone**

**Other:**

**57.Which of the following scenarios is most recognizable to you?***

***Mark only one oval.***

**The person I care for falls (regularly)** ***Skip to question 58***

**The person I care for is experiencing nighttime restlessness** ***Skip to question 64***

**A**

**fall**

**The person I care for is experiencing agitation (stress/irritability) *Skip* *to question 71***

**None of the above scenarios are recognizable to me** ***Skip to question 77***

**It's Monday morning, around 11 a.m. the smart box has detected a fall. You are informed by the platform that your loved one has fallen and is on the bathroom floor.**

**https://docs.google.com/forms/d/18Pt5mtN4vZZkLIT3Xir3FLLfwuD5lzI3PSXlo3DSzIQ/edit** **20/38**

**25/04/2022, 11:49** **Questionnaire**

**58.What do you think of this situation?***

***Mark only one oval.***

**An emergency**

**Urgent, but not an emergency**

**Normal situation**

**59.How would you like to be informed by the smart box in the event that your loved one has fallen?***

***Check all that apply.***

**A notification or pop-up, such as Caren's. A**

**text message/SMS**

**A (priority) email**

**A phone call or voicemail**

**An update on the platform so I can check for myself when I have time**

**Other:**

**60.Should the box send you a notification when your loved one has fallen?***

***Mark only one oval.***

**Yes, right away**

**Yes, after a few minutes if my neighbor has not risen**  **Yes, but only at certain times that I set myself**

**No, I'll check myself as soon as I have time**

**https://docs.google.com/forms/d/18Pt5mtN4vZZkLIT3Xir3FLLfwuD5lzI3PSXlo3DSzIQ/edit** **21/38**

**25/04/2022, 11:49** **Questionnaire**

**61.Who should receive a message when your loved one has fallen?***

***Mark only one oval.***

**Only I myself need to receive a notification**

**Only all informal carers (e.g. family, friends or neighbours) must receive a notification**

**Only the care providers (for example nurse, home care or therapist) must receive a notification**

**All informal carers and caregivers must receive a notification**

**Only myself and the care providers (for example nurse, home care or therapist) must receive a notification**

**Only myself and the caregivers (e.g. family, friends or neighbours) must receive a notification**

**Not applicable**

**62.What should the smart box do if you do not immediately (within a minute) open the notification if your loved one has fallen?***

***Mark only one oval.***

**Please wait 5 minutes so that I have time to view the notification**

**Contact another carer of my loved one**

**Contact my loved one's healthcare providers**

**Call the GP/112**

**Other:**

**https://docs.google.com/forms/d/18Pt5mtN4vZZkLIT3Xir3FLLfwuD5lzI3PSXlo3DSzIQ/edit** **22/38**

**25/04/2022, 11:49** **Questionnaire**

**63.If the smart box informs me about the fall, I would prefer to receive this information as follows:***

***Mark only one oval.***

**Raw data: the information received by the smart box, without any interpretation.**

**See the speech bubble above!**

**Interpreted data: the information from the smart box is immediately analyzed by the box, then the information is shared by the platform. See the speech bubble above!**

**Interpretation and Suggestion: The information is instantly analyzed by the smart box, along with the information, a suggestion is made for what action you can take. See the speech bubble above!**

**Other:**

***Skip to question 77***

**nocturnal**

**unrest**

**You have had your loved one on the phone at 1 am in which he/she told you that he/she has trouble sleeping. At 3 am, the smart box observes that your loved one is constantly turning in bed, getting out of bed and wandering around the house.**

**https://docs.google.com/forms/d/18Pt5mtN4vZZkLIT3Xir3FLLfwuD5lzI3PSXlo3DSzIQ/edit** **23/38**

**25/04/2022, 11:49** **Questionnaire**

**64.What do you think of this situation?***

***Mark only one oval.***

**An emergency**

**Urgent, but not an emergency**

**Normal situation**

**65.In what way would you like to be informed by the smart box in case your loved one has nightly restlessness?***

***Check all that apply.***

**A notification or pop-up, such as Caren's. A**

**text message/SMS**

**A (priority) email**

**A phone call or voicemail**

**An update on the platform so I can check for myself when I have time**

**Other:**

**66.Would you like to receive a notification every time your loved one gets out of bed?***

***Mark only one oval.***

**Yes**

**No**

**I want to be able to adjust this option myself**

**67.Would you like to receive a notification every time your next of kin goes to bed?***

***Mark only one oval.***

**Yes**

**No**

**I want to be able to adjust this option myself**

**https://docs.google.com/forms/d/18Pt5mtN4vZZkLIT3Xir3FLLfwuD5lzI3PSXlo3DSzIQ/edit** **24/38**

**25/04/2022, 11:49** **Questionnaire**

**68.Would you like to receive a detailed report on your loved one's nightly restlessness?**

*****

***Mark only one oval.***

**Yes, every day**

**Yes, observe for a few days and send a report if the unrest continues.**

**Yes, observe for a few weeks and send a report if the turmoil continues.**

**No, send this information to the healthcare providers**

**Other:**

**69.What should the box do if you do not open the notification immediately (within a minute) if your loved one shows nocturnal restlessness?***

***Mark only one oval.***

**Please wait 5 minutes so that I have time to view the notification**

**Contact another carer of my loved one**

**Contact my loved one's healthcare providers**

**Call the GP/112**

**Other:**

**https://docs.google.com/forms/d/18Pt5mtN4vZZkLIT3Xir3FLLfwuD5lzI3PSXlo3DSzIQ/edit** **25/38**

**25/04/2022, 11:49** **Questionnaire**

**70.If the box informs me about the nightly unrest, I would prefer to receive this information as follows***

***Mark only one oval.***

**Raw data: the information received by the smart box, without any interpretation.**

**See the speech bubble above!**

**Interpreted data: the information from the smart box is immediately analyzed by the box, then the information is shared by the platform. See the speech bubble above!**

**Interpretation and Suggestion: The information is instantly analyzed by the smart box, along with the information, a suggestion is made for what action you can take. See the speech bubble above!**

**Other:**

***Skip to question 77***

**Agitation**

**(stress or**

**irritability**

**during the day)**

**The smart box increasingly detects that your loved one is restless and irritable during the day. For example, he/she throws the newspaper on the floor, kicks the table leg or reacts angrily when the bus stops at the stop in front of the house.**

**https://docs.google.com/forms/d/18Pt5mtN4vZZkLIT3Xir3FLLfwuD5lzI3PSXlo3DSzIQ/edit** **26/38**

**25/04/2022, 11:49** **Questionnaire**

**71.What do you think of this situation?***

***Mark only one oval.***

**An emergency**

**Urgent, but not an emergency**

**Normal situation**

**72.In what way would you like to be informed by the smart box if your loved one shows restlessness or irritability during the day?***

***Check all that apply.***

**A notification or pop-up, such as Caren's. A**

**text message/SMS**

**A (priority) email**

**A phone call or voicemail**

**An update on the platform so I can check for myself when I have time**

**Other:**

**73.When would you like to receive this information:**

***Mark only one oval.***

**At any time of the day**

**Only at certain times, which I have indicated myself**

**Never**

**https://docs.google.com/forms/d/18Pt5mtN4vZZkLIT3Xir3FLLfwuD5lzI3PSXlo3DSzIQ/edit** **27/38**

**25/04/2022, 11:49** **Questionnaire**

**74.Would you like to receive a detailed report on the restlessness or irritability of your loved one? Yes, every day***

***Mark only one oval.***

**Yes, every day**

**Yes, observe for a few days and send a report if the unrest continues.**

**Yes, observe for a few weeks and send a report if the turmoil continues.**

**No, send this information to the healthcare providers**

**Other:**

**75.What should the box do if you do not open the notification immediately (within a minute) if your loved one shows restlessness or irritability?***

***Mark only one oval.***

**Please wait 5 minutes so that I have time to view the notification**

**Contact another carer of my loved one**

**Contact my loved one's healthcare providers**

**Call the GP/112**

**Other:**

**https://docs.google.com/forms/d/18Pt5mtN4vZZkLIT3Xir3FLLfwuD5lzI3PSXlo3DSzIQ/edit** **28/38**

**25/04/2022, 11:49** **Questionnaire**

**76.If the box informs me about stress or irritability during the day, I would prefer to receive this information as follows.***

***Mark only one oval.***

**Raw data: the information received by the smart box, without any interpretation.**

**See the speech bubble above!**

**Interpreted data: the information from the smart box is immediately analyzed by the box, then the information is shared by the platform. See the speech bubble above!**

**Interpretation and Suggestion: The information is instantly analyzed by the smart box, along with the information, a suggestion is made for what action you can take. See the speech bubble above!**

**Other:**

***Skip to question 77***

**Normal**

**day**

**It is a normal day in which the smart box has not observed anything abnormal. Your loved one has had a healthy day in which he has eaten, exercised and slept sufficiently.**

**https://docs.google.com/forms/d/18Pt5mtN4vZZkLIT3Xir3FLLfwuD5lzI3PSXlo3DSzIQ/edit** **29/38**

**25/04/2022, 11:49** **Questionnaire**

**77.How would you like to be informed by the smart box if your loved one has a normal day?***

***Check all that apply.***

**A notification or pop-up, such as Caren's. A**

**text message/SMS**

**A (priority) email**

**A phone call or voicemail**

**An update on the platform so I can check for myself when I have time**

**Other:**

**78.When would you like to receive this information?***

***Mark only one oval.***

**At any time of the day**

**Only at certain times, which I have indicated myself**

**Never**

**79.How often would you like to receive an update?***

***Mark only one oval.***

**I want to receive an update after every activity (slept, ate, exercised)**

**Send me the information at the end of the day**

**I do not need to receive information about this**

**I'll check the platform myself as soon as I have time**

**I would like to be able to fill in what information I receive and when**

**Other:**

**https://docs.google.com/forms/d/18Pt5mtN4vZZkLIT3Xir3FLLfwuD5lzI3PSXlo3DSzIQ/edit** **30/38**

**25/04/2022, 11:49** **Questionnaire**

**80.What should the smart box do if you do not immediately (within a minute) open the notification that your loved one is having a normal day?***

***Mark only one oval.***

**Please wait 5 minutes so that I have time to view the notification**

**Contact another carer of my loved one**

**Contact my loved one's healthcare providers**

**Call the GP/112**

**Other:**

**81.If the smart box informs me about a normal day, I would prefer to receive this information as follows***

***Mark only one oval.***

**Raw data: the information received by the smart box, without any interpretation.**

**See the speech bubble above!**

**Interpreted data: the information from the smart box is immediately analyzed by the box, then the information is shared by the platform. See the speech bubble above!**

**Interpretation and Suggestion: The information is instantly analyzed by the smart box, along with the information, a suggestion is made for what action you can take. See the speech bubble above!**

**Other:**

**https://docs.google.com/forms/d/18Pt5m tN4vZZkLIT3Xir3FLLfwuD5lzI3PSXlo3DSzIQ/edit** **31/38**

**25/04/2022, 11:49**

**Technology**

**acceptance**

**Questionnaire**

**Keep the aforementioned smart box in combination with a communication platform as a product in mind when answering the following questions.**

**Please indicate to what extent you agree with the following statements:**

**82.Using this product would make my caregiving easier.***

***Mark only one oval.***

**Totally disagree**

**Disagree**

**Partly disagree**

**Neutral (don't disagree/don't agree)**

**Partly agree**

**Agree**

**Totally agree**

**83.Using this product would enable me to provide the care my loved one needs.***

***Mark only one oval.***

**Totally disagree**

**Disagree**

**Partly disagree**

**Neutral (don't disagree/don't agree)**

**Partly agree**

**Agree**

**Totally agree**

**https://docs.google.com/forms/d/18Pt5mtN4vZZkLIT3Xir3FLLfwuD5lzI3PSXlo3DSzIQ/edit** **32/38**

**25/04/2022, 11:49** **Questionnaire**

**84.Using this product would increase my effectiveness in taking care of my loved one.***

***Mark only one oval.***

**Totally disagree**

**Disagree**

**Partly disagree**

**Neutral (don't disagree/don't agree)**

**Partly agree**

**Agree**

**Totally agree**

**85.I would find this product helpful in caring for my loved one.***

***Mark only one oval.***

**Totally disagree**

**Disagree**

**Partly disagree**

**Neutral (don't disagree/don't agree)**

**Partly agree**

**Agree**

**Totally agree**

**86.Learning how to use this product would be easy for me.***

***Mark only one oval.***

**Totally disagree**

**Disagree**

**Partly disagree**

**Neutral (don't disagree/don't agree)**

**Partly agree**

**Agree**

**Totally agree**

**https://docs.google.com/forms/d/18Pt5mtN4vZZkLIT3Xir3FLLfwuD5lzI3PSXlo3DSzIQ/edit** **33/38**

**25/04/2022, 11:49** **Questionnaire**

**87.I would find this product easy to use.***

***Mark only one oval.***

**Totally disagree**

**Disagree**

**Partly disagree**

**Neutral (don't disagree/don't agree)**

**Partly agree**

**Agree**

**Totally agree**

**88.If I had access to this product, I would start using it in caring for my loved one.***

***Mark only one oval.***

**Totally disagree**

**Disagree**

**Partly disagree**

**Neutral (don't disagree/don't agree)**

**Partly agree**

**Agree**

**Totally agree**

**https://docs.google.com/forms/d/18Pt5mtN4vZZkLIT3Xir3FLLfwuD5lzI3PSXlo3DSzIQ/edit** **34/38**

**25/04/2022, 11:49** **Questionnaire**

**89.What is your highest obtained diploma? (optional)**

***Mark only one oval.***

**No degree**

**Primary education**

**VMBO, MBO 1-2**

**HAVO, VWO, HBS, MBO 3-4**

**Bachelor (HBO/WO)**

**Master's degree (HBO/WO)**

**Other:**

**90.Would you like to participate in a next study about the provision of care?***

***Mark only one oval.***

**Yes**

**No**

**Maybe**

**Contact details - Caregiver**

***Skip to question 91***

***Skip to question 91***

**Please enter your email address here, or if we may call you your telephone number, so that we can contact you.**

**91.Enter your email address here**

**92.Enter your telephone number here**

**Questions for healthcare providers**

**https://docs.google.com/forms/d/18Pt5mtN4vZZkLIT3Xir3FLLfwuD5lzI3PSXlo3DSzIQ/edit** **35/38**
