# Supplementary material for: Exploring the Needs and Requirements of Informal Caregivers of Older Adults With Cognitive Impairment From Sensor-Based Care Solutions: Multimethod Study
Source: JMIR Aging. 2023 Oct 25;6:e49319. doi: 10.2196/49319 (PMC10632915; doi:10.2196/49319)
Supplement: Multimedia Appendix 2 [file aging_v6i1e49319_app2.docx]

**Appendix 2: Interview Guide**

| Introduction | - Welcome and purpose of the study - Research Aim - Data collection and use - Structure of the interview - Informed consent form - Other instructions |
| --- | --- |
| Background | - Age - Profession - Relationship with the care recipient - Age of care recipient - Care duration - Cognitive impairment stage and diagnosis - Motivation for providing care - Living situation of care recipient (same house, somewhere else) - Care hours per week - Care responsibilities - Involved other informal - Formal caregivers and their care responsibilities - Visit of formal caregivers - Care collaboration and communication with healthcare providers - Care collaboration: advantage and disadvantages |
| Current experience with technology | - Do you use any technology currently (names) in care? - How do the communication with current sensing technology takes place? - What is your experience with this system/technology? (both positive and negative) |
| Future expectations | Do you experience specific concerns about some situations when your loved one is alone at home? And why?   - Prioritize situations - When do these situations occur? - If such a situation arises now, what will you do? - In these situations, what is going well and what is not going well in current care from the perspective of used technology? - What could help you in such a situation? What would you like to know? - What information are you missing? And that information is important? |
| Proposed unobtrusive sensing solution | - Explanation on working of Wi-Fi Channel state information-based sensing solution.   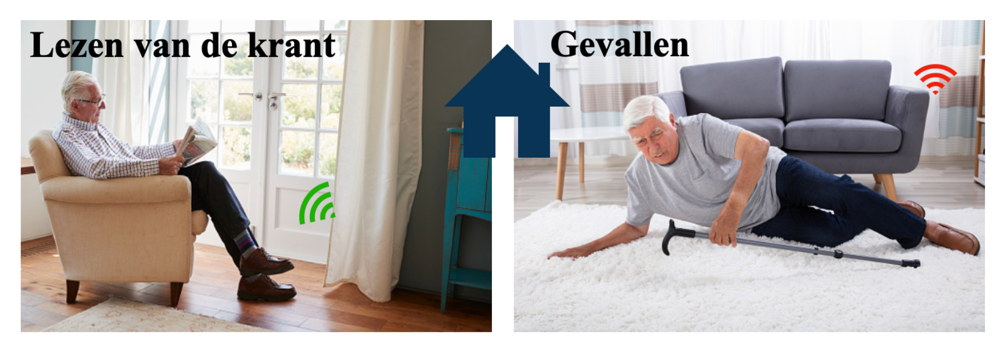  We are researching on a new unobtrusive sensing technology to recognize various care situations in older adult care. It is unobtrusive, which means that your loved one does not have to wear anything on the body and does not require line of sight to be able to use this device. As you can see in the image, a small sensing box, can be placed in the corner of the house. This smart box can track various activities and observe important changes, such as drinking or eating less, change in heart rate or breathing, nocturnal unrest, but also emergencies such as a fall. The system can learn to recognize certain situations and inform you as a caregiver about them. In order to develop this system, we therefore want to understand what your needs are and requirements from such as system. Specifically, we want to ask you which situations are most important to you? when would you like to receive information from the platform? what exactly do you want to know? how and when you want to know. |
| Information platform (oral explanation) | 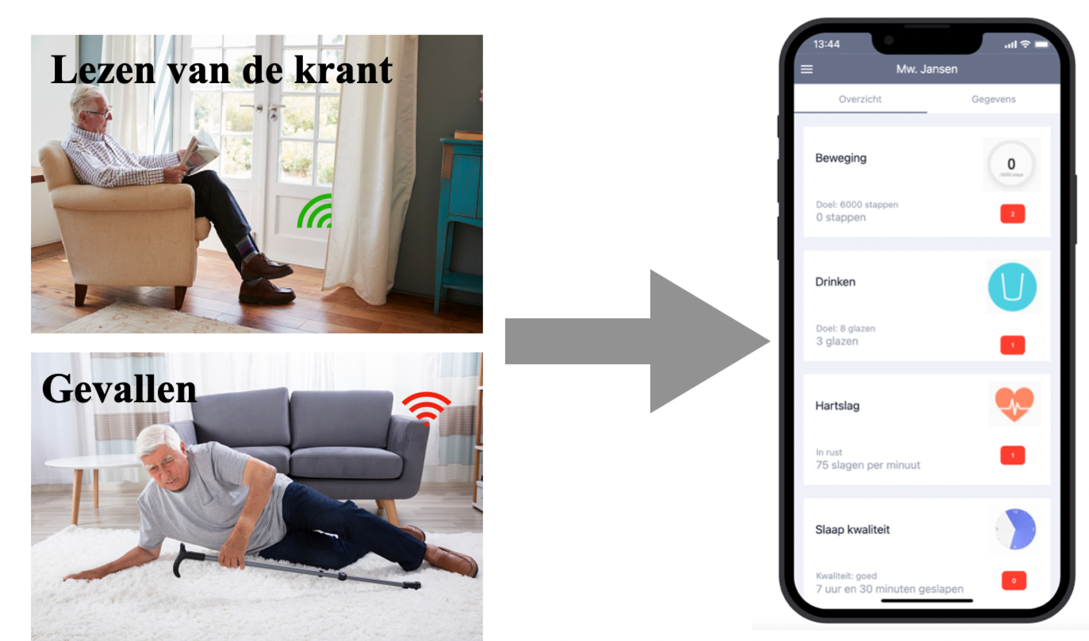 |
| Acceptance and IC needs  (Situation based question (choose one situation):   - Fall - Nocturnal unrest - Agitation   Parallel questions on Normal daily life ) | - What would you think if such a system were used to care for your loved one? Advantages and disadvantages - How could this communication platform support you in the care you provide? W.r.t. situation chosen. - What would you like to know / what information would you like to receive? How detailed should the information be? - What should the information look like? (now vs future, fact vs advice, current vs prediction, measurement vs interpretation) - How would you like to receive this information? App/SMS/phone call/popup/ email. Why? - When would you like to be informed about this situation? - Who should receive the notifications? How should the transfer of information take place? - What information should be shared and what information should not? With whom? By whom? How? (Other informal carers and healthcare professionals) - What do you think of the addition of additional social aspects? For example, do you think that you can share experiences via the platform? Or receive advice? - Interaction with others? (How do you want to have contact) - Connection with other caregivers or professionals, why or why not? |
| Design conditions for the platform and system | - What information should be visible immediately after opening the app/website? Should emergencies be among them? - What do you expect from the information presentation/layout of the platform? What should it look like? How would you present the information? - What do you think a report should look like? (color, text, sound, voice .. ) - What should the platform be able to do? What should it all offer? - What would you prefer an app or a website? Why? - On which device would you like to use the platform? - What do you want to be able to adjust in the platform? - For example, the moment that notifications are sent, how long after the situation occurs, to whom? - What should platform do in case of emergency - What do you think if the system sends you a reminder if you don't respond in case of an emergency? |
| System credibility (trust, false alarms, security, and privacy) | - To what extent would you trust such a system? And what is important for you to be able to have confidence in this system? - What needs to be done to maintain this trust? - What difference does it make if the system indicates how certain it is of an observation? - How does this trust affect your use of the system? - How could this (or a similar) system support you in ensuring the safety of your loved one? - How do you see security and privacy? Which is more important and why? - What are your views on false alarms? Does that change your confidence in the system? |
| Future Usage, feedback, compliments, suggestions | - How will the use of this system change your care responsibilities? To what extent will it improve care? - In which areas does the system have added value? - What would you need to start using it? - What do you need to continue using the system? - Would you recommend it to other caregivers? - Would you have money for it? - Would you like to help improve the system by giving it feedback by clicking yes/no about the accuracy of the report after notifications? - Would you like system to give you feedback about how you acted in a particular situation? - What it be useful if the platform gives you a compliment or feedback based on how you acted in a particular care situation? |
| End questions | - Is there anything else you'd like to add/discuss/suggest that hasn't been asked yet? |

*Please note that this guide has been translated from Dutch to English.*
